# Supramolecular Assembly and Chirality of Synthetic Carbohydrate Materials

**DOI:** 10.1002/anie.202008153

**Published:** 2020-10-07

**Authors:** Soeun Gim, Giulio Fittolani, Yoshiharu Nishiyama, Peter H. Seeberger, Yu Ogawa, Martina Delbianco

**Affiliations:** ^1^ Department of Biomolecular Systems Max-Planck-Institute of Colloids and Interfaces Am Mühlenberg 1 14476 Potsdam Germany; ^2^ Department of Chemistry and Biochemistry Freie Universität Berlin Arnimallee 22 14195 Berlin Germany; ^3^ Univ. Grenoble Alpes CNRS CERMAV 38000 Grenoble France

**Keywords:** carbohydrates, chirality, microED, self-assembly, structure-property correlations

## Abstract

Hierarchical carbohydrate architectures serve multiple roles in nature. Hardly any correlations between the carbohydrate chemical structures and the material properties are available due to the lack of standards and suitable analytic techniques. Therefore, designer carbohydrate materials remain highly unexplored, as compared to peptides and nucleic acids. A synthetic d‐glucose disaccharide, **DD**, was chosen as a model to explore carbohydrate materials. Microcrystal electron diffraction (MicroED), optimized for oligosaccharides, revealed that **DD** assembled into highly crystalline left‐handed helical fibers. The supramolecular architecture was correlated to the local crystal organization, allowing for the design of the enantiomeric right‐handed fibers, based on the l‐glucose disaccharide, **LL**, or flat lamellae, based on the racemic mixture. Tunable morphologies and mechanical properties suggest the potential of carbohydrate materials for nanotechnology applications.

## Graphical abstract

Synthetic oligosaccharides and microED analysis enable the molecular level characterization of carbohydrate materials. The local crystal organization is correlated to the supramolecular architecture, allowing for the design of helical structures as well as flat lamellae.

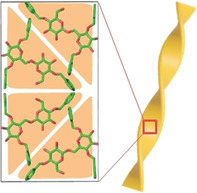

## Introduction

Nature is based on self‐assembling systems, resulting in highly complex and dynamic architectures.^[1]^ Peptides, nucleic acids, and carbohydrates can form ordered hierarchical structures based on the synergistic effect of different non‐covalent interactions, such as van der Waals, electrostatic, π‐π stacking, hydrophobic interactions, as well as hydrogen and coordination bonds.^[2]^ Much work has been devoted to gain a molecular description of these natural systems. Models have been developed to reduce nature's complexity, allowing for a better description and manipulation. Di‐phenylalanine (**FF**) was identified as minimal repeating unit of the amyloid fibrils involved in the Alzheimer's disease progression.^[3]^ This system permitted to unveil mechanistic processes of amyloid formation and to design novel inhibitors.^[4]^ Due to its ability to self‐assemble into several geometries, this simple dipeptide found hundreds of applications in nanotechnology.^[5]^ Similarly, the discovery of particular peptide and nucleic acid sequences, able to generate stable aggregates, prompted the development of artificial analogues with tunable shapes and properties.^[6]^

Carbohydrates, the most abundant organic material on Earth, are also capable of forming hierarchical architectures.^[7]^ Still, their molecular level description remains limited due to difficult access to pure materials and a lack of suitable analytical techniques.^[8]^ The potential of carbohydrate materials remains thus highly underexploited. The synthesis of well‐defined polysaccharide sequences is labor intensive. Moreover, the intrinsic flexibility of short oligosaccharides has hampered their use for the formation of supramolecular structures.^[9]^ As a consequence, carbohydrates have found limited applications in nanotechnology, with the only exception being nanocellulose.^[10]^ Still, nanocellulose is often directly extracted from natural sources (top‐down approach), limiting molecular design opportunities and detailed structure‐function correlations.^[11]^

**Figure 1:**
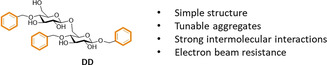
Chemical structure and features of the d‐glucose disaccharide **DD**.

Recently, we discovered that simple synthetic oligosaccharides can self‐assemble in different morphologies.^[12]^ These systems could offer a new bottom‐up approach to understand and exploit carbohydrate materials. We have identified the disaccharide **DD** (Figure 1) as ideal model to study molecular self‐assembly in polysaccharides. This compound offers several advantages to develop analytical methods that can be translated to the study of natural carbohydrate materials. **DD** i) is easy to synthesize, ii) can form tunable supramolecular structures, contains aromatic functionalities that iii) stabilize the self‐assembly and iv) make it less susceptible to electron beam irradiation allowing for electron microscopy (EM) analysis.^[13]^ These aspects enabled the development of assays to probe the molecular structures and the chirality of the aggregates. Key interactions that drive the self‐assembly were uncovered, suggesting insights into the mechanism of formation.

## Results and Discussion

**DD** self‐assembles, upon solvent switch, into fibers that are micrometers long and nanometers wide (Supporting Information). Fiber formation occurs almost instantaneously upon injection of a **DD** stock solution (100 mg mL^−1^ in hexafluoroisopropanol HFIP) into water, to reach a final concentration of 2 mg mL^−1^. The crystal structure and the molecular packing were investigated with X‐ray diffraction (XRD), solid‐state nuclear magnetic resonance (ssNMR) spectroscopy, and microcrystal electron diffraction (MicroED). MicroED became extremely popular for structural determination since it directly reveals the molecular organization of the self‐assembled structure in its native state, reducing tedious crystallization trials that can alter the supramolecular organization.^[14]^ Nanoscale structural heterogeneities of molecular solids can be characterized due to the small electron probe size.^[15]^ To date, MicroED has been employed rarely to study simple oligosaccharides due to their sensitivity to the electron beam. **DD** is an ideal substrate to develop MicroED, since the benzyl groups present in the molecule render it more resistant to prolonged irradiation.^[13]^

**Figure 2:**
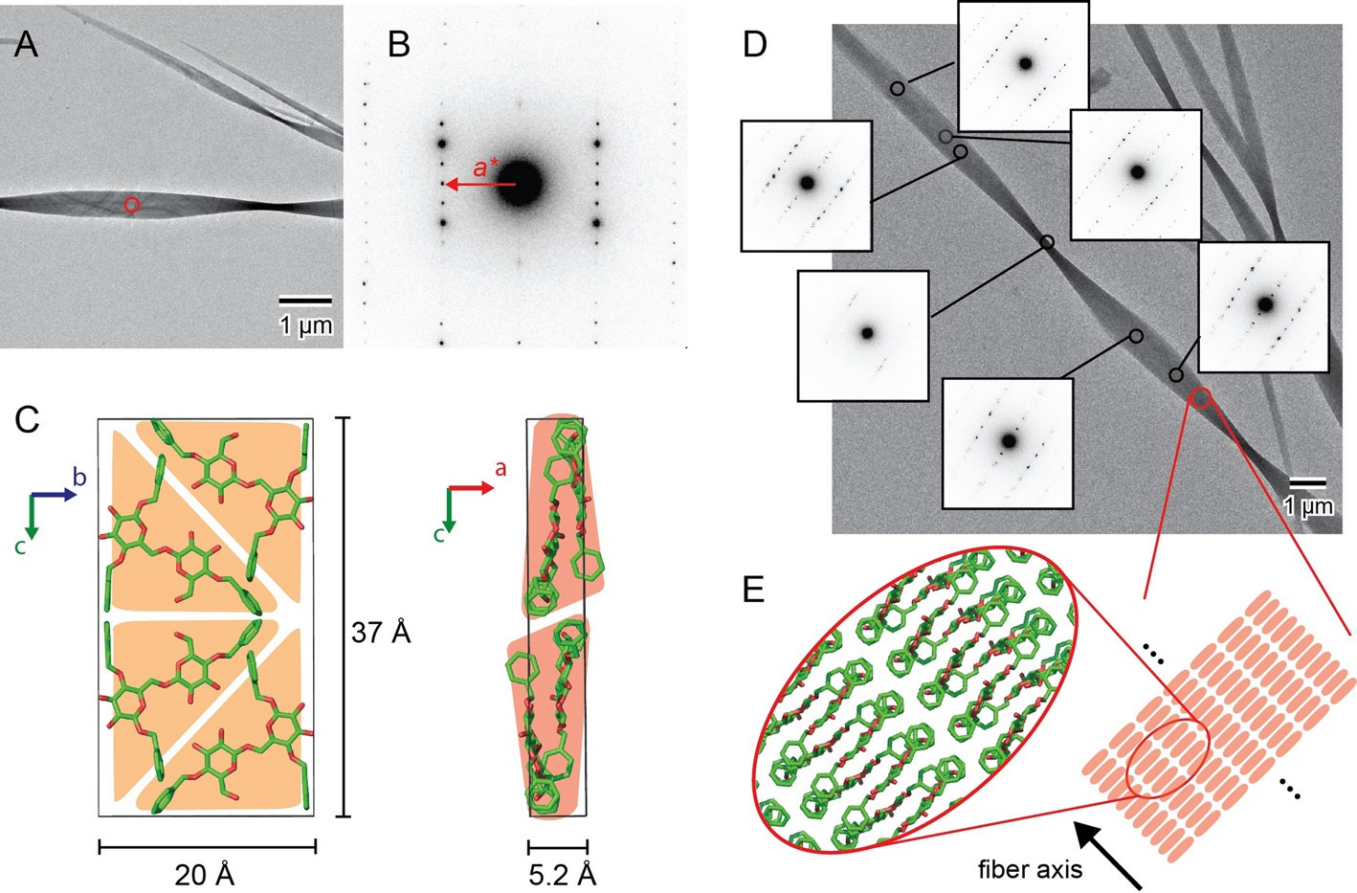
MicroED analysis of self‐assembled **DD** performed at cryogenic temperature. A) Diffraction contrast image of **DD** crystals. B) Electron diffraction diagram obtained from the circled area in (A). C) Tentative molecular packing model of **DD** in the unit cell determined from tilt‐series MicroED experiments. Hydrogen atoms are removed for clarity. D) Twist geometry followed by sequential electron microdiffraction. E) Schematic of molecular packing manner in the fibrillar **DD** crystal.

XRD shows the high crystallinity of the assemblies (Supporting Information, Figure S3) and ssNMR indicates the presence of two sets of **DD** in a single unit cell (Figure S1). MicroED analysis on **DD** crystals was performed at cryogenic temperature (Figure 2). Electron diffraction patterns obtained from the flat part (circled area in Figure 2 A) provided spot diffraction patterns with a resolution of about 1.2 Å, indicating its single crystal nature and high crystallinity. The structure was determined based on a tilt series MicroED analysis as an orthorhombic unit cell with *a*=5.2 Å, *b*=20 Å, *c*=37 Å (Figure S2; Figure 2 C). The reflection positions calculated based on the ED analysis are in general agreement with those in the powder X‐ray diffraction profile (Figure S3). The calculated reflection positions are lower angle shifted due to a slight overestimation of unit cell dimensions by the ED analysis, as previously demonstrated for native cellulose crystals.^[16]^ The unit cell contains four **DD** molecules, giving a density of 1.06 g cm^−3^. The tentative molecular packing model in *bc* and *ac* projections (Figure 2 C) show a short *a*‐axis, indicating that **DD** molecules assume an overall flat conformation and stack along the *a*‐axis. The glucose ring planes are oriented roughly in the *bc* plane. The aromatic rings assemble in a close proximity to each other. The interactions between the aromatic rings are mostly C−H⋅⋅⋅π type edge‐to‐face interactions. No face‐to‐face π‐π stacking is present in the packing model, as the molecular spacing in the stacking direction (5.2 Å, *a*‐axis), is larger than the maximum acceptable distance for π‐π stacking formation (3.8 Å).^[17]^ The carbohydrate moieties are not in close contact with each other. The relatively low density of the crystal implies that water molecules may be involved in the crystalline lattice. The distances between hydroxyl groups of adjacent molecules allow forming water‐bridged hydrogen bonds with a single water molecule between the hydroxyl groups. Further refinement will permit to explicitly determine the presence of water as well as the hydrogen bonding network in the crystal structure.

In addition to crystallographic information, MicroED can provide structural insight into larger scale supramolecular structures, such as a twist. Ready correlation of local molecular organization with supramolecular assembly could revolutionize the description of supramolecular systems based on small molecules, but has been hardly exploited.^[18]^ The **DD** crystals were subjected to a sequential electron microdiffraction experiment with an electron probe size of about 100 nm. Each ED pattern obtained along the fiber axis corresponds to a different lattice projection (Figure 2 D), revealing left‐handed twists along their fiber axes. In all ED patterns, the a*‐axis is oriented along the fiber axis of the crystal, indicating that the stacking of flat molecular sheets happens parallel to the fiber axis (Figure 2 E). The crystal twists along the stacking direction, implying that this supramolecular twist is likely to originate from a slight rotation between the stacked molecules. The apparent half twist pitch (180 degree rotation) is about 5 μm in most crystallites, resulting in a rotation per unit cell of about 0.02 degree. While crystal twists were observed previously for natural carbohydrate crystals such as cellulose and chitin, the mechanism of twisting of carbohydrate crystals is still elusive.[^11b^, ^19^] These results suggests that well‐defined synthetic systems could shine light on the twisting mechanism of natural systems as well as on the relationship between molecular chirality and supramolecular structures.

The system is tunable and different morphologies are observed when the synthesis is performed at different temperatures (Figure S5). When the self‐assembly is performed at high temperature (75 °C), large flat fibers (width in the μm range) are formed. Those fibers become shorter and thinner (width <0.5 μm) with a narrow distribution as the assembly temperature is decreased (Figure S6). Electron and X‐ray diffraction analysis show the same pattern for the three samples, confirming identical crystalline structure, but different fibril dimensions (Figures S5 and S7). The helical pitch can be controlled adjusting the assembly conditions, with a longer pitch observed when the assembly is performed with a higher content of organic solvent (Figure S8). A slower formation rate is observed.

**Figure 3:**
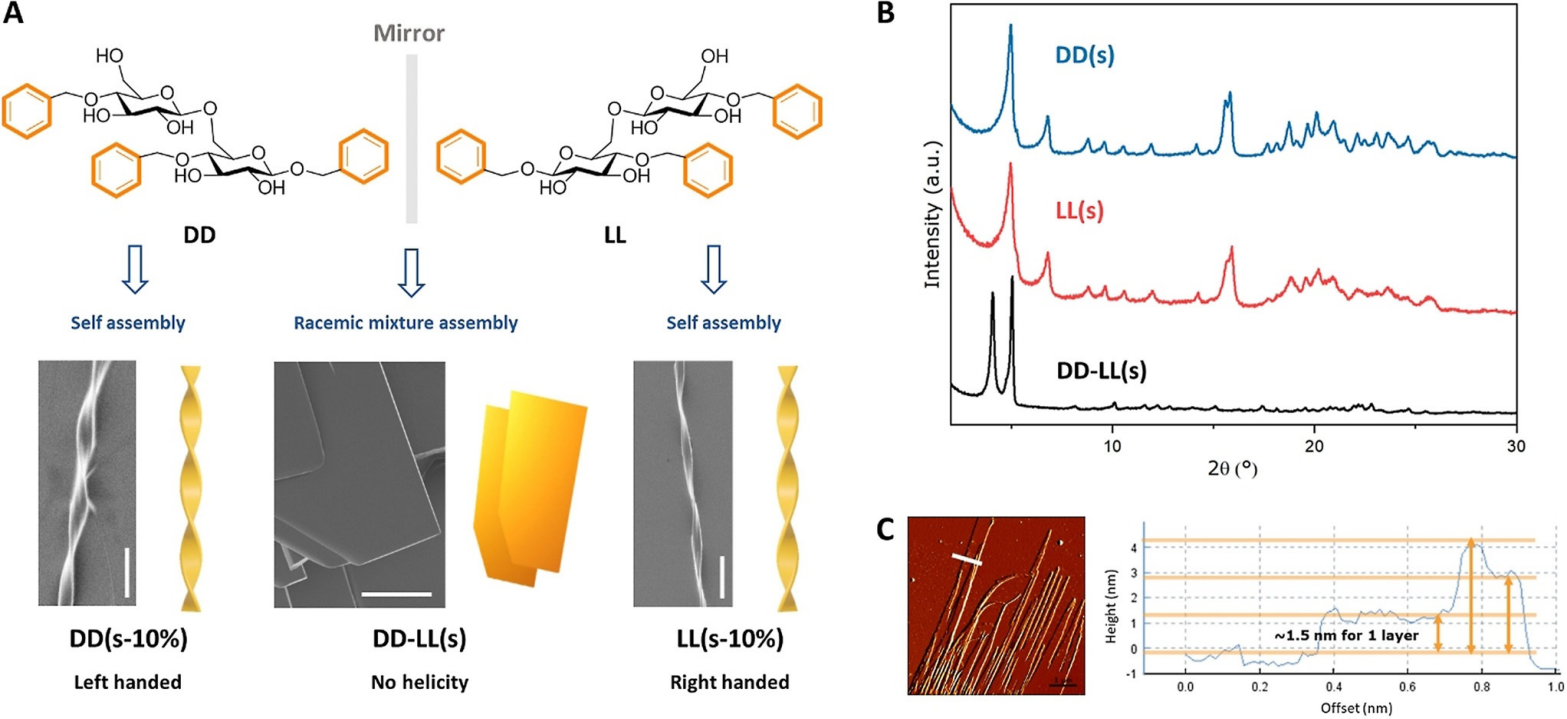
A) Chemical structures of the **DD** and **LL** enantiomers and SEM images of their supramolecular assembly. While **DD(s‐10 %)** (left) and **LL(s‐10 %)** (right) show the opposite supramolecular chirality, the racemic mixture **DD‐LL(s)** (middle) generates a flat sheet‐like structure (scale bars: 10 μm). B) XRD spectra for **DD(s)** (blue), **LL(s)** (red), and **DD‐LL(s)** (black). C) AFM image and cross‐sectional analysis of **DD‐LL(s)**. The sample names indicate the disaccharide (e.g., **DD**), the sample preparation method (e.g., **s**), and the content of HFIP in water (e.g., **10 %**). Terminology definition: **DD(s‐10 %)** means the compound **DD** prepared by solvent‐switch method with 10 % HFIP. If the content of HFIP is not mentioned, the standard content is 2 %.

The helicity of the fibers offers an additional tool to tune the properties of self‐assembled materials.^[20]^ Indeed, chirality is an important design mode in peptide nanotechnology.^[21]^ In particular, heterochiral peptide‐based systems offer many advantages such as increased stiffness of the self‐assembled fibers,^[22]^ increased stability towards enzymatic degradation,^[23]^ and access to new morphologies.^[24]^ Inspired by this work, the enantiomeric disaccharide (**LL**) was synthesized starting from l‐glucose (Supporting Information). Upon solvent switch, **LL** forms the enantiomeric helical fibers (right handed), confirming the direct correlation between oligosaccharide chirality and fiber helicity (Figure 3 A; Figure S11). To the best of our knowledge, this is the first observation of oligosaccharide molecular chirality governing supramolecular chirality. Synthetic enantiomeric oligosaccharides will become powerful tools to establish correlations between polysaccharide chirality and assembly. The racemic mixture **DD‐LL(s)** aggregates in a completely new and flat geometry (Figure 3 A; Figure S10). XRD confirmed that both enantiomers, **DD(s)** and **LL(s)**, have identical crystallinity, whereas the racemic mixture packs in a different manner (Figure 3 B), as previously observed for heterochiral peptides assemblies.^[24]^ Alteration of the 1:1 ratio between **DD** and **LL** creates irregularity in the structure, likely composed of flat structures and helical fibers (Figure S13). AFM analysis of the flat aggregates (**DD‐LL(s)**) suggests that the two enantiomers may construct a layer‐by‐layer supramolecular assembly (Figure 3 C). The height of the sheets varies from a few hundred nanometers to several micrometers, with the single layer measuring 1.5 nm, which is comparable to one dimension of the disaccharide (Figure S4). This may indicate that the disaccharides in the racemic mixture align laterally forming a single layer, as previously observed for heterochiral peptide assemblies.^[25]^ The material, resulting from the stacking of multiple layers, shows a Young's modulus of 2.029±0.093 GPa (Figure S12).

The possibility of performing self‐assembly directly on a 2D surface is attractive, as it can generate films with controlled morphologies.^[26]^ Such designer surfaces have found applications^[27]^ in catalysis,^[28]^ as semiconducting materials,^[29]^ as chemical sensors,^[30]^ and as optical devices.^[31]^ Additionally, monitoring the assembly on a two dimensional surface could give insights into the assembly mechanism due to the slower nucleation and crystallization rate at the interface.^[32]^

**Figure 4:**
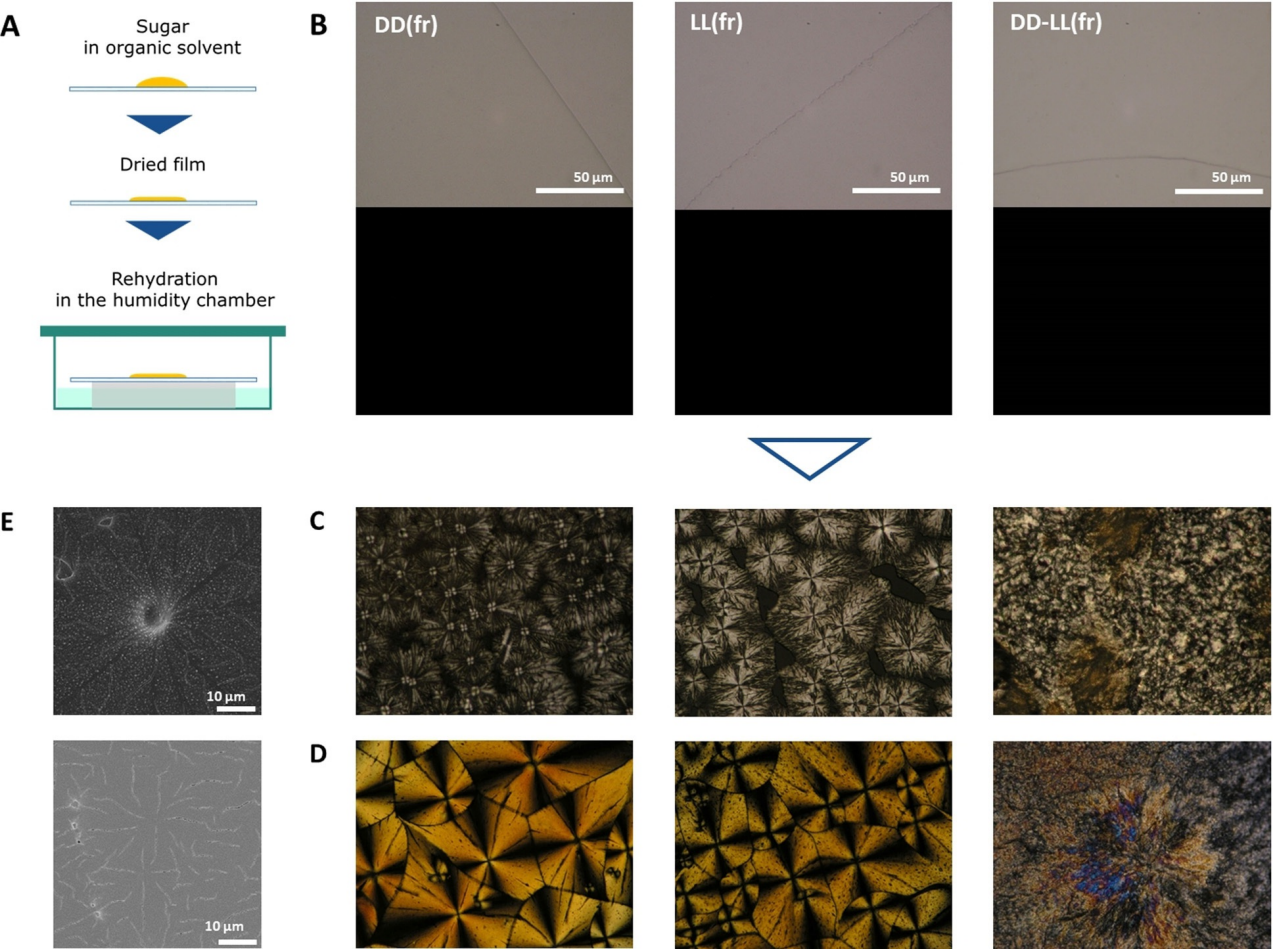
A) Cartoon illustrating the film‐rehydration (fr) method. B–D) POM images of **DD(fr)**, **LL(fr)**, and **DD‐LL(fr)** at time 0 (B) and after 3 h (center (C) and boundary (D) of the film). E) SEM images of **DD(fr)** (top, center; bottom, boundary). **DD(fr)** and **LL(fr)** show identical patterns. Crystallization produces the classical Maltese cross pattern. Different parts of the film show slightly different patterns, likely due to small differences in local concentration (Figure S15). The core shows complete separation of each spherulite (C), whereas the boundary shows densely connected spherulites (D). Both enantiomerically pure samples produce (**DD(fr)** and **LL(fr)**) spherulites, whereas the racemic mixture **DD‐LL(fr)** does not show any defined pattern. The sample names indicate the disaccharide (e.g., **DD**) and the sample preparation method (e.g., **fr**); as an example, **DD(fr)** means the compound **DD** prepared by film‐rehydration method. If the concentration is not mentioned, the standard concentration is 100 mg mL^−1^.

A continuous film was generated upon drop casting of a HFIP solution of **DD** (Figure S14). This highly hydrophobic film transformed into a fibrous structure, upon contact with water (Figure S14). The 2D self‐assembly was repeated, incubating the drop‐cast film in a humidity chamber with saturated vapor (Figure 4 A). Dewetting of thin film using an anti‐solvent is a common procedure to generate particular morphologies on surfaces and relies on the spontaneous surface diffusion and organization of the material.^[33]^ The assembly progression was monitored with polarized optical microscopy (POM). Three samples were prepared from solutions of **DD**, **LL** and the racemic mixture **DD‐LL**. The drop‐cast films are amorphous, resulting in black background when observed between crossed polarizers (Figure 4 B). Upon hydration (3 h), the films obtained from **DD** and **LL** develop spherulites composed of a nucleation center and multiple lamellae growing from the core (Figures 4 C,D). The racemic mixture **DD‐LL** does not form any defined structure. The XRD profile of the **DD** film at *t*=0 confirms its amorphous nature; sharp peaks develop upon hydration, indicating crystallization (Figure S15). The 2D assembly is concentration dependent (Figure 4 E; Figures S16 and S17).

**Figure 5:**
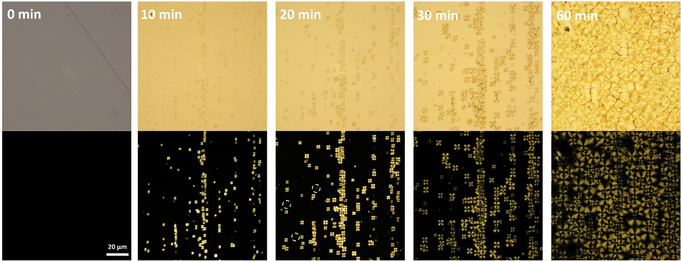
POM images of **DD(fr)** at 0, 10, 20, 30, and 60 min observed between parallel (top) and crossed (bottom) polarizers. White circles represent additional nucleation observed after 20 min.

The nucleation process can be monitored in real time, offering the opportunity to explore crystallization kinetics (Figure 5). Spherulitic growth is common for synthetic polymers,^[34]^ but not for carbohydrates, as sugar crystallization is often shock‐induced and kinetics are too fast to follow.^[35]^ Upon hydration, the amorphous film starts to nucleate, developing highly organized spherulites showing the classical Maltese cross (when observed between cross polarizers). The interaction of the molecules in the film with the water vapor (hydrophobic interaction) triggers a structural reorganization and promotes the assembly of **DD** into a fibrous structure. The fibers grow radially from the nucleation core, giving rise to highly organized morphologies, until they encounter an adjacent spherulite (Figure S18). Additional nucleation is observed during the crystallization process (Figure 5, white circles). The radius of the spherulites doubles every 10 minutes. The transition from amorphous to crystallized state is completed within 3 h. During this transition, the mechanical properties of the film are drastically affected. Nanoindentation was performed to measure the stiffness of the thin film, **DD(fr)**, before and after exposure to water vapor (Figure S19). The amorphous film has a Young's modulus of 2.047±0.060 GPa. After vapor‐induced crystallization, the film shows a 3‐fold increased stiffness, 6.072±1.429 GPa.

## Conclusion

We have established a model system to study carbohydrate materials. The strong intermolecular interactions and electron beam resistance in disaccharide **DD** enabled the development of assays to study polysaccharide materials at the molecular level. A MicroED analysis based on a tilt‐series ED acquisition was, for the first time, applied to an oligosaccharide system, permitting the reconstruction of the crystal unit of the assembled materials in their native state. This method is key to the structural analysis of crystalline carbohydrate systems. Since most oligo‐ and polysaccharides crystallize into nano‐ to micrometer‐sized crystallites, MicroED, optimized for electron sensitive materials, is an important tool to understand the structural and conformational diversity of carbohydrates. Moreover, the local crystal organization can be correlated to the larger supramolecular architecture, offering insights into self‐assembly.

The supramolecular fibers showed a distinct helicity that could be correlated to the molecular lever chirality, offering a new mode to tune the supramolecular structure. For instance, pitch and helicity could be modulated adjusting the assembly conditions or the chemical composition of the self‐assembling solution. Helical structures (left or right handed) as well as flat lamellae could be obtained on demand. Similar synthetic approaches will help to correlate polysaccharide chirality and their assembly.

Self‐assembly could be performed in solution or on 2D surfaces, resulting in highly tunable and versatile materials. Two‐dimensional spherulites could be generated from simple disaccharides under mild conditions to produce carbohydrate surfaces with tunable physical and mechanical properties. We anticipate that these findings will have applications in carbohydrate nanotechnology, just as the discovery of the di‐phenylalanine **FF** self‐assembly stimulated various follow‐up applications.[^5c^, ^16b^, ^36^]

## Conflict of interest

The authors declare no conflict of interest.

## Supporting information

As a service to our authors and readers, this journal provides supporting information supplied by the authors. Such materials are peer reviewed and may be re‐organized for online delivery, but are not copy‐edited or typeset. Technical support issues arising from supporting information (other than missing files) should be addressed to the authors.

Supplementary
