# Supplementary material for: Supramolecular Assembly and Chirality of Synthetic Carbohydrate Materials
Source: Angew Chem Int Ed Engl. 2020 Oct 7;59(50):22577–83. doi: 10.1002/anie.202008153 (PMC7756587; doi:10.1002/anie.202008153)
Supplement: Supplementary file 1 — Supplementary [file ANIE-59-22577-s001.pdf]

Angewandte Chemie
Eine Zeitschrift der Gesellschaft Deutscher Chemiker

Supporting Information

Supramolecular Assembly and Chirality of Synthetic Carbohydrate Materials

*Soeun Gim, Giulio Fittolani, Yoshiharu Nishiyama, Peter H. Seeberger, Yu Ogawa, and Martina Delbianco**

anie_202008153_sm_miscellaneous_information.pdf

1. Table of Contents

1. Table of Contents 1
2. General materials and methods 2
3. Synthesis 3
3.1. Synthesis of L-Glc building block 3
Synthesis of 2,3,4,6-tetra-O-acetyl-1-O-acetyl-L-glucopyranoside, **2** 3
Synthesis of ethyl 2,3,4,6-tetra-O-acetyl-1-thio-β-L-glucopyranoside, **3** 6
Synthesis of ethyl 4,6-O-benzylidene-1-thio-β-L-glucopyranoside, **4** 9
Synthesis of ethyl 2,3-di-O-benzoyl-4,6-O-benzylidene-1-thio-β-L-glucopyranoside, **5** 12
Synthesis of ethyl 2,3-di-O-benzoyl-4-O-benzyl-1-thio-β-L-glucopyranoside, **6** 15
Synthesis of ethyl 2,3-di-O-benzoyl-4-O-benzyl-6-O-(9-fluorenylmethoxycarbonyl)-1-thio-β-L-glucopyranoside, **7** 18
3.2. Synthesis of LL 21
Synthesis of benzyl 2,3-O-dibenzoyl-4-O-benzyl-β-L-glucopyranoside, **8** 21
Synthesis of **LL** 24
4. Self-Assembly 27
5. NMR analysis 29
6. MicroED analysis 30
7. Morphological analysis at different T 32
8. Chirality analysis 34
9. 2D self-assembly 37
10. References 41

2. General materials and methods

All chemicals used were reagent grade and used as supplied unless otherwise noted. Analytical thin-layer chromatography (TLC) was performed on Merck silica gel 60 F254 plates (0.25 mm). Compounds were visualized by UV irradiation or dipping the plate in a staining solution (sugar stain: 10% H_2SO_4 in EtOH; CAM: 48 g/L ammonium molybdate, 60 g/L ceric ammonium molybdate in 6% H_2SO_4 aqueous solution). Flash column chromatography was carried out by using forced flow of the indicated solvent on Fluka Kieselgel 60 M (0.04 – 0.063 mm). Analysis and purification by normal and reverse phase HPLC was performed by using an Agilent 1200 series. Products were lyophilized using a Christ Alpha 2-4 LD plus freeze dryer. ^{1}H, ^{13}C, HSQC, and COSY NMR spectra were recorded on a Varian 400-MR (400 MHz), Varian 600-MR (600 MHz), or Bruker Biospin AVANCE700 (700 MHz) spectrometer. Spectra were recorded in $CDCl_3$ by using the solvent residual peak chemical shift as the internal standard ($CDCl_3$: 7.26 ppm ^{1}H, 77.0 ppm ^{13}C), in D_2O using the solvent as the internal standard (D_2O: 4.79 ppm ^{1}H) or in MeOD using the solvent as the internal standard (MeOD: 4.87 ppm ^{1}H, 49.0 ppm ^{13}C). The ^{13}C cross-polaization/ magic angle spinning (CP/MAS) NMR spectrum was measured with a Bruker Avance II spectrometer operating at 100 MHz for ^{13}C. The dry powder sample was packed in a zirconia rotor with a diameter of 3 mm. The measurement was performed with a spinning speed of 12 kHz, a sweep width of 29761 Hz, a recycle delay of 2 s and a cross-polarization contact of 2 ms. The ^{13}C chemical shifts were calibrated with the glycine carboxyl group at 176.03 ppm. High resolution mass spectra were obtained using a 6210 ESI-TOF mass spectrometer (Agilent). ESI mass spectra were run on IonSpec Ultima instruments. IR spectra were recorded on a Perkin-Elmer 1600 ATR-FTIR spectrometer. Optical rotations were measured by using a Perkin-Elmer 241 and Unipol L1000 polarimeter.

Scanning electron microscopy (SEM) images were obtained with a Gemini SEM, LEO 1550 system with cold field emission gun operation at 3 kV. All the samples were coated with Au/Pd. Atomic force microscopy (AFM) was carried out with a Multimode Nanoscope IIIa AFM. Images were attained with conventional AC mode and flattened without further modification. Qualitative imaging (QI) mode was applied for nanoindentation with a silicon cantilever. With JPK data processing software, force-distance curves were fit to the Hertz model and manipulated to obtain Young's modulus. Transition in ordered status was verified via polarized optical microscope (POM), an Olympus BX41, and X-ray diffractometer (XRD), a Bruker D8 with Cu Kα radiation. Circular dichroism (CD) spectra were acquired with a Chrascan qCD spectrometer (Applied Photophysics Ltd. Leatherhead, UK) using a quartz cuvette (Hellma GmbH & Co. KG, Mullheim, Germany) at RT with a band width of 1 nm. FT-IR spectra were recorded using a Perkin Elmer FT-IR spectrometer, Spectrum 100.

Transmission electron microscopy was performed using a JEM-2100Plus (JEOL Ltd., Japan) equipped with a GATAN Rio16 CMOS camera, operated at an accelerating voltage at 200 kV. Drops of aqueous suspensions of crystallites were deposited on glow-discharged carbon-coated copper grids. All the measurements were carried out at a cryogenic temperature with an Elsa cryo-transfer holder (Gatan Inc., USA) to protect the electron sensitive **DD** crystals. All electron micrographs and electron diffraction patterns were recorded on a Gatan Rio 16 camera (Gatan Inc., USA). Low-dose bright-field imaging and microED (µED) measurements were achieved using the SerialEM program. The tilt-series experiments were performed with an increment angle of 0.1° and an overall rotation of about 40°. A selected area aperture with a diameter of 200 nm was inserted. A focused electron probe with a diameter of about 100 nm was used to follow the twist geometry of **DD** crystals. The camera length was calibrated using a powder ED pattern of evaporated aluminum. The µED patterns were analyzed using the Fiji program and in-house scripts. The tilt-series ED patterns were remapped in reciprocal space to determine the unit cell parameters (Fig. S2)

The 3D cartoons in Figure 3A were made with Blender v2.82.

3. Synthesis

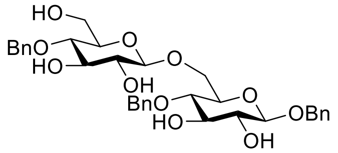

DD

Compound **DD** was synthesized according to a previously reported procedure.[1]

3.1. Synthesis of L-Glc building block

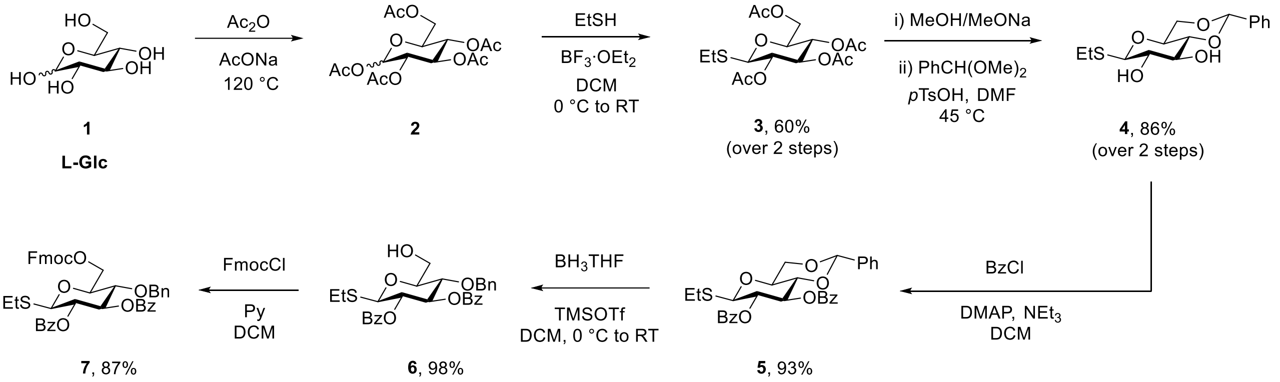

Scheme S1. Synthetic steps for the synthesis of L-Glc building block

Synthesis of 2,3,4,6-tetra-O-acetyl-1-O-acetyl-L-glucopyranoside, **2**

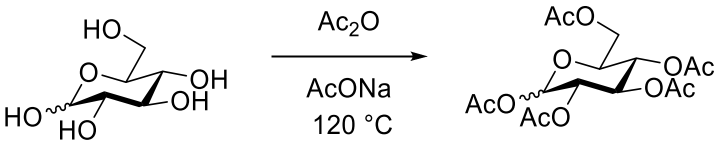

1 **2**

L-glucose **1** (4.95 g, 27.5 mmol) was slowly added (over 10 min) to a stirred solution of sodium acetate (1.9 g, 13.8 mmol) in acetic anhydride (45 mL) at 120°C. The mixture was stirred for 1 h and then cooled to RT. The reaction was quenched with ice (100 g) and diluted with EtOAc. The reaction mixture was washed five times with saturated aqueous solution of $NaHCO_3$ and one time with brine. The crude was passed through a short plug of silica with EtOAc, dried over Na_2SO_4, and concentrated *in vacuo*. 2,3,4,6-tetra-*O*-acetyl-1-*O*-acetyl-L-glucopyranoside **2** was obtained as a white solid (11.3 g, quantitative yield, α:β ratio 1:4).

^{1}H NMR (400 MHz, Chloroform-*d*) δ 6.32 (dd, *J* = 3.7, 1.2 Hz, 0.2H), 5.71 (dd, *J* = 8.3, 1.0 Hz, 0.8H), 5.52 – 5.40 (m, 0.2H), 5.26 (ddd, *J* = 18.8, 9.9, 0.9 Hz, 1H), 5.18 – 5.06 (m, 2H), 4.33 – 4.23 (m, 1H), 4.15 – 4.05 (m, 1H), 3.83 (ddd, *J* = 9.9, 4.6, 2.2 Hz, 0.8H), 2.14 – 1.98 (m, 15H). ^{13}C NMR (101 MHz, Chloroform-*d*) δ 170.81, 170.78, 170.40, 170.27, 169.82, 169.54, 169.41, 169.13, 168.93, 91.78, 89.16, 72.88, 72.80, 70.28, 69.91, 69.27, 67.93, 67.78, 61.52, 21.04, 20.98, 20.86, 20.82, 20.72, 20.61. $[\alpha]_D^{20}$ -20.56 (c 1.00 g/100 mL, $CHCl_3$). IR v = 2927, 1752, 1370, 1216, 1039 cm^{-1}. (ESI-HRMS) m/z 413.099 $[M+Na]^+$ ($C_{16}H_{22}O_{11}$ requires 413.105).

^{1}H NMR of 2 (400 MHz, $CDCl_3$)

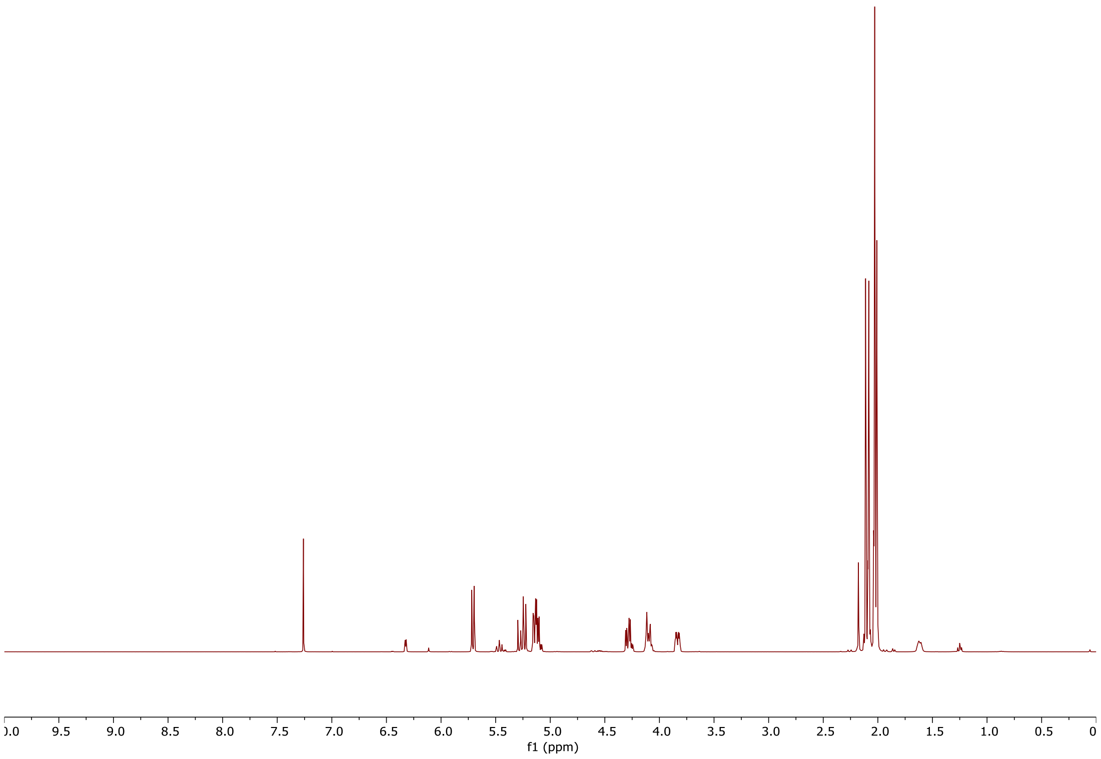

^{13}C NMR of 2 (101 MHz, $CDCl_3$)

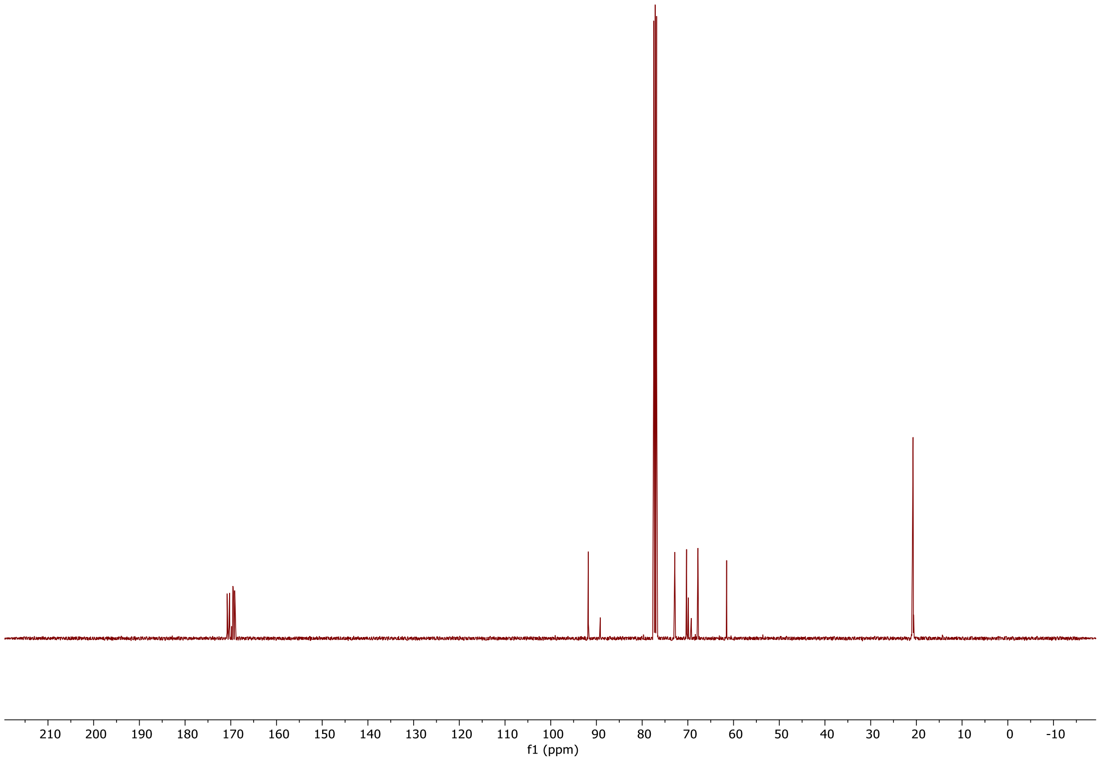

HSQC NMR of 2 ($CDCl_3$)

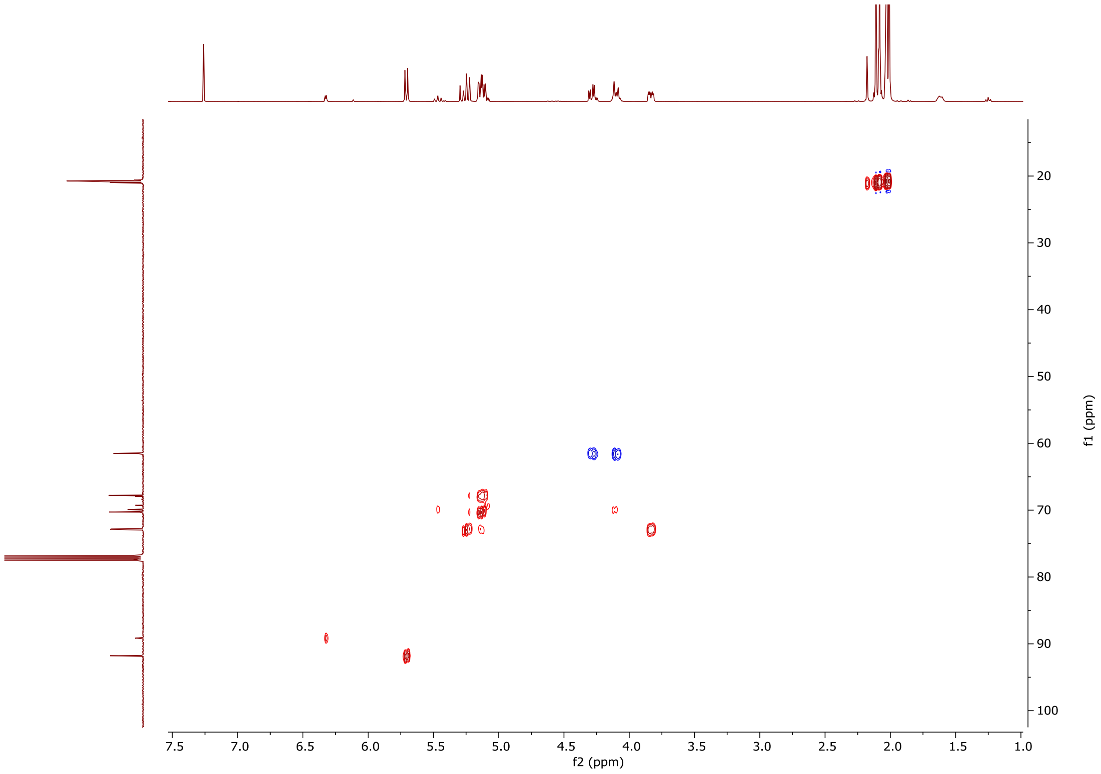

COSY NMR of 2 ($CDCl_3$)

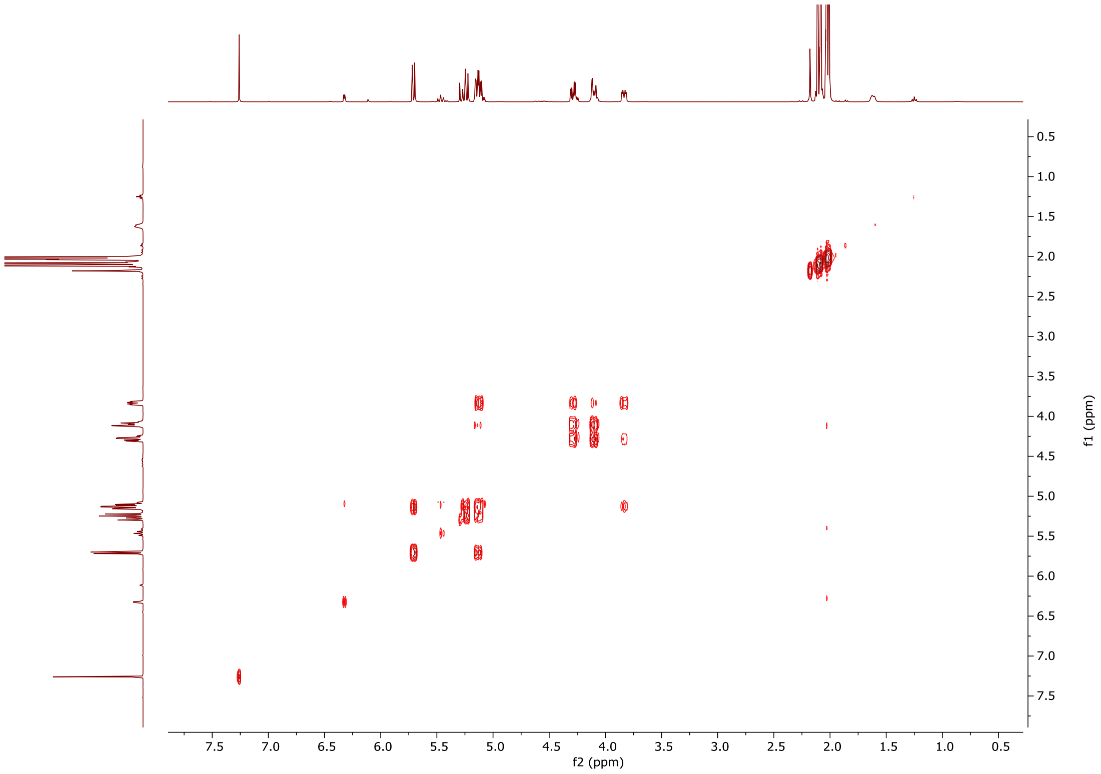

Synthesis of ethyl 2,3,4,6-tetra-O-acetyl-1-thio-β-L-glucopyranoside, **3**

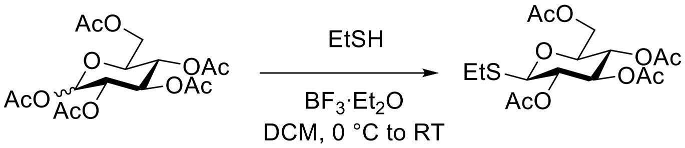

2 **3**

$BF_3{\cdot}EtO_2$ complex (5.4 mL, 42.6 mmol) was slowly added to a stirred solution of 2,3,4,6-tetra-*O*-acetyl-1-*O*-acetyl-L-glucopyranoside **2** (11.34 g, 29.1 mmol) with EtSH (5.4 mL, 73.1 mmol) in DCM (100 mL) at 0 °C under Ar atmosphere. The reaction was slowly brought to RT and, after 2 h, quenched with saturated aqueous solution of $NaHCO_3$ at 0 °C. The mixture was diluted with DCM and washed three times with saturated aqueous solution of $NaHCO_3$ and once with brine. The organic phase was dried over Na_2SO_4 and concentrated under reduced pressure. The crude product was purified by flash column chromatography (hexane:EtOAc = 4:1→2:1) to yield **3** as a white solid (6.78 g, 60%).
^{1}H NMR (400 MHz, Chloroform-*d*) δ 5.22 (t, *J* = 9.4 Hz, 1H), 5.12 – 5.00 (m, 2H), 4.49 (d, *J* = 10.0 Hz, 1H), 4.24 (dd, *J* = 12.4, 4.9 Hz, 1H), 4.13 (dd, *J* = 12.4, 2.4 Hz, 1H), 3.71 (ddd, *J* = 10.0, 4.9, 2.3 Hz, 1H), 2.79 – 2.62 (m, 2H), 2.10 – 1.98 (m, 12H), 1.27 (t, *J* = 7.4 Hz, 3H). ^{13}C NMR (101 MHz, Chloroform-*d*) δ 170.84, 170.38, 169.58, 169.58, 83.66, 82.49, 75.98, 74.02, 69.92, 68.42, 24.33, 20.90, 20.89, 20.78, 20.75, 14.96. $[\alpha]_D^{20}$ 20.24 (c 0.42 g/100 mL, $CHCl_3$). IR *v* = 1752, 1370, 1223, 1038 cm^{-1}. (ESI-HRMS) m/z 415.098 $[M+Na]^+$ ($C_{16}H_{24}O_9SNa$ requires 415.103).

^{1}H NMR of 3 (400 MHz, CDCl$_3$)

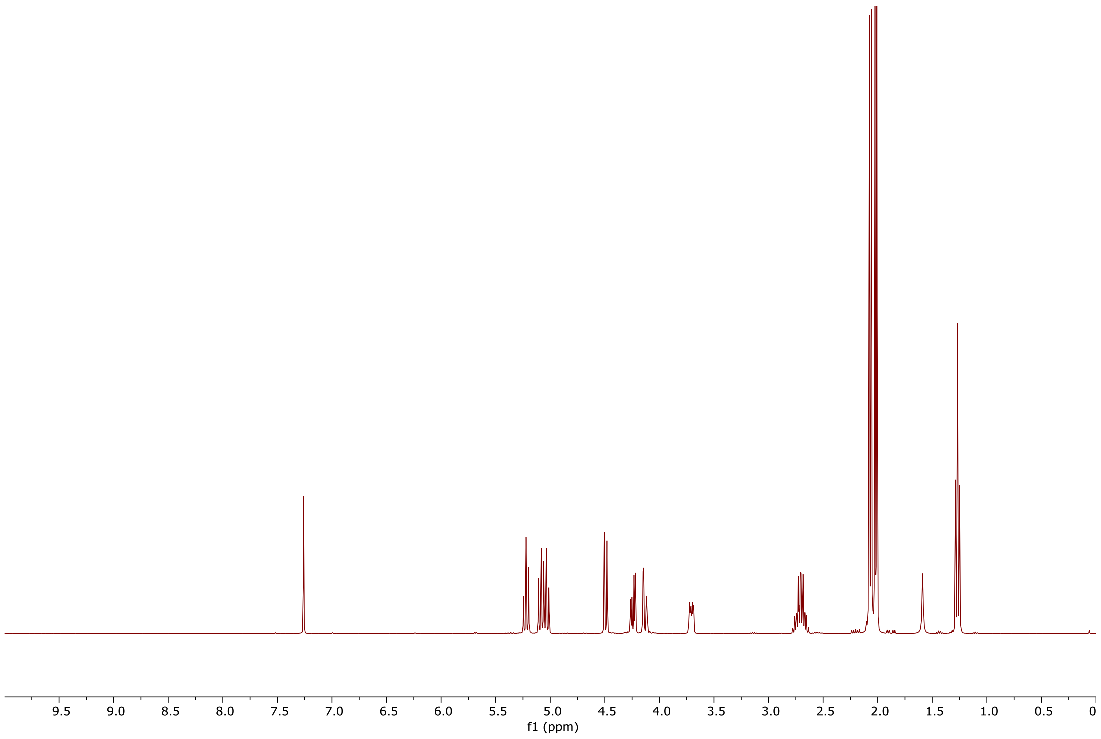

^{13}C NMR of 3 (101 MHz, CDCl$_3$)

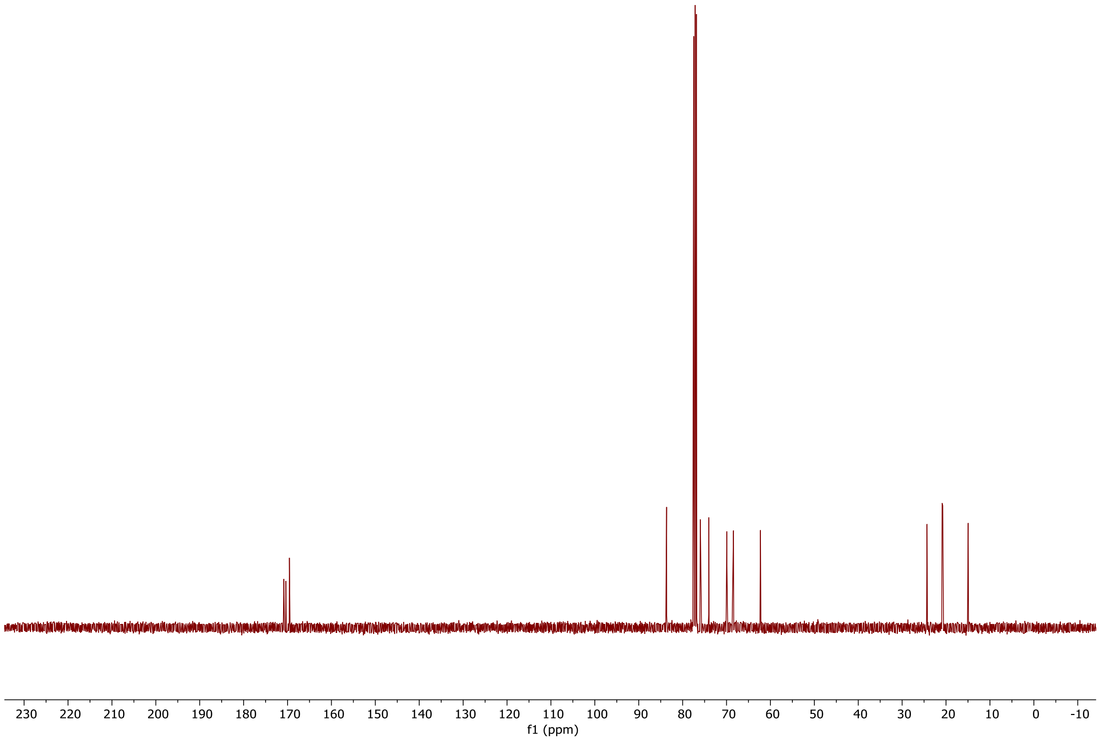

HSQC NMR of 3 ($CDCl_3$)

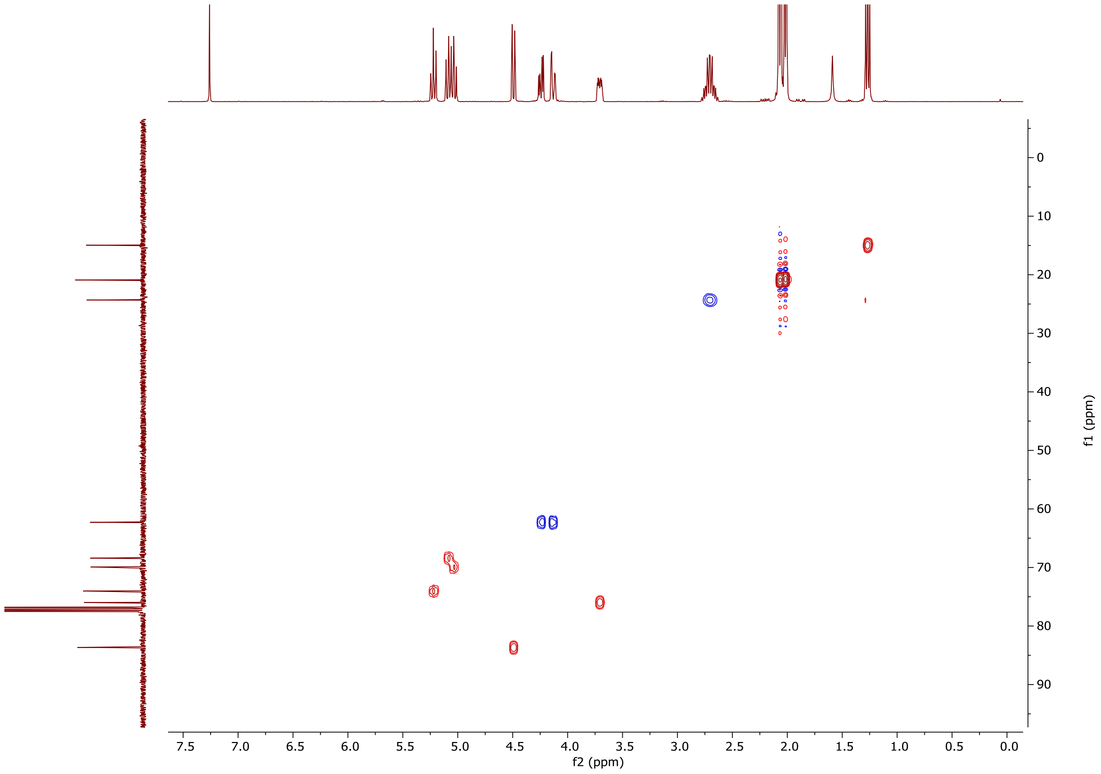

COSY NMR of 3 ($CDCl_3$)

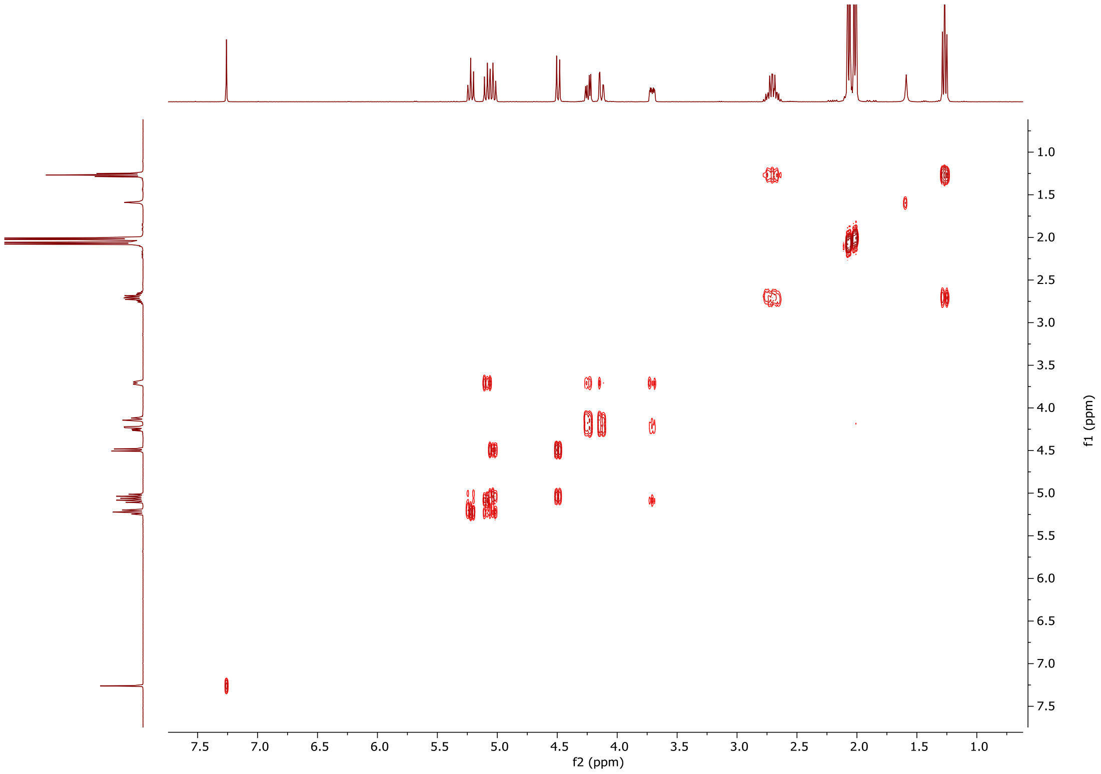

Synthesis of ethyl 4,6-O-benzylidene-1-thio-β-L-glucopyranoside, **4**

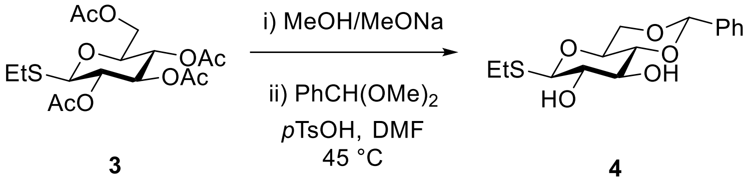

2,3,4,6-tetra-*O*-acetyl-1-*O*-acetyl-L-glucopyranoside **2** (6.78 g, 17.3 mmol) was dissolved in MeOH (60 mL) and a 0.5 M solution of MeONa in MeOH (7.0 mL, 3.5 mmol) was slowly added. The mixture was stirred at RT for 1 h. The reaction was neutralized with Amberlite IR-120 (H^+ form), filtered, and concentrated under reduced pressure. The product was used in the next step without any further purification assuming quantitative conversion. The crude product was dissolved in DMF and a catalytic amount of *p*-toluensulfonic acid monohydrate (356 mg, 1.7 mmol) was added to the mixture. Benzaldehyde dimethyl acetal (5.2 mL, 34.6 mmol) was then added dropwise at RT to the stirred solution. The reaction was heated to 45°C overnight, after which time it was quenched with triethylamine (2 mL) at 0 °C. The reaction mixture was diluted with EtOAc and washed three times with saturated aqueous solution of $NaHCO_3$ and once with brine. The organic layer was dried over Na_2SO_4 and concentrated under reduced pressure. The sticky oil was recrystallized from hexane:EtOAc to yield **4** as a white solid (4.65 g, 86% over 2 steps).
^{1}H NMR (400 MHz, Chloroform-*d*) δ 7.53 – 7.45 (m, 2H), 7.42 – 7.33 (m, 3H), 5.55 (s, 1H), 4.47 (d, *J* = 9.8 Hz, 1H), 4.36 (dd, *J* = 10.5, 4.8 Hz, 1H), 3.85 (t, *J* = 8.8 Hz, 1H), 3.81 – 3.74 (m, 1H), 3.59 (t, *J* = 9.2 Hz, 1H), 3.55 – 3.48 (m, 2H), 2.91 – 2.66 (m, 2H), 1.33 (t, *J* = 7.4 Hz, 3H). ^{13}C NMR (101 MHz, Chloroform-*d*) δ136.96, 129.49, 128.52, 126.40, 102.08, 86.78, 80.47, 74.65, 73.28, 70.68, 68.72, 24.97, 15.47. $[\alpha]_D^{20}$ 48.95 (c 0.39 g/100 mL, $CHCl_3$). IR *v* = 3406, 2926, 1074, 1029, 1004, 700 cm^{-1}. (ESI-HRMS) m/z 335.083 $[M+Na]^+$ ($C_{15}H_{20}O_5SNa$ requires 335.092).

^{1}H NMR of 4 (400 MHz, $CDCl_3$)

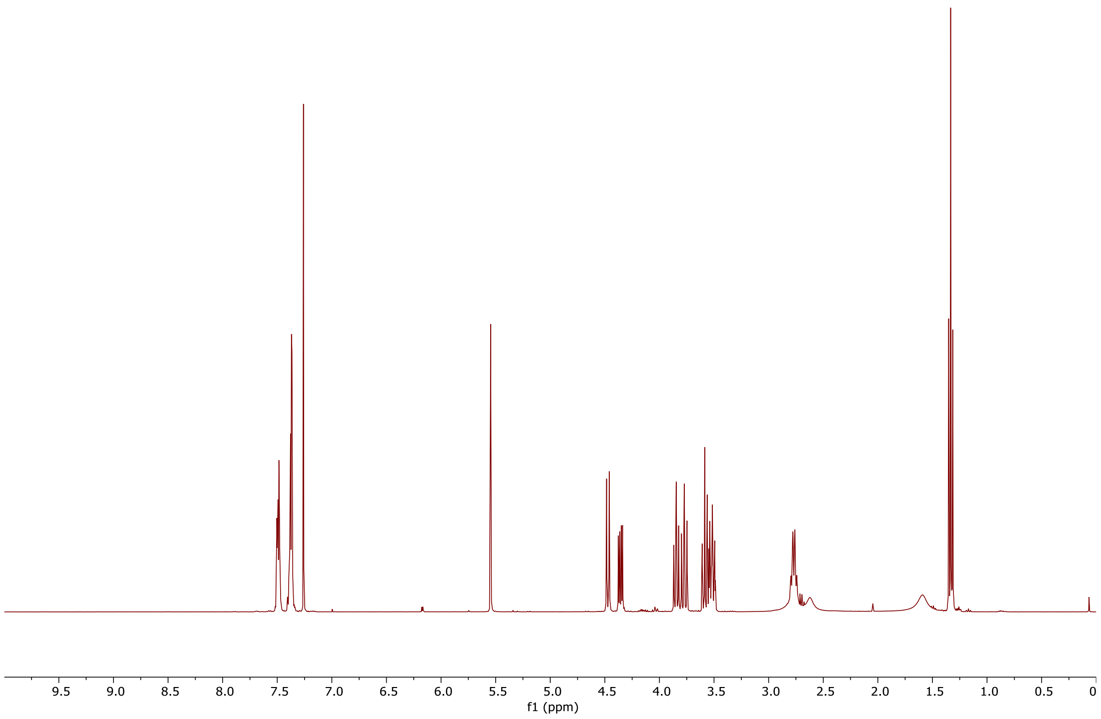

^{13}C NMR of 4 (101 MHz, $CDCl_3$)

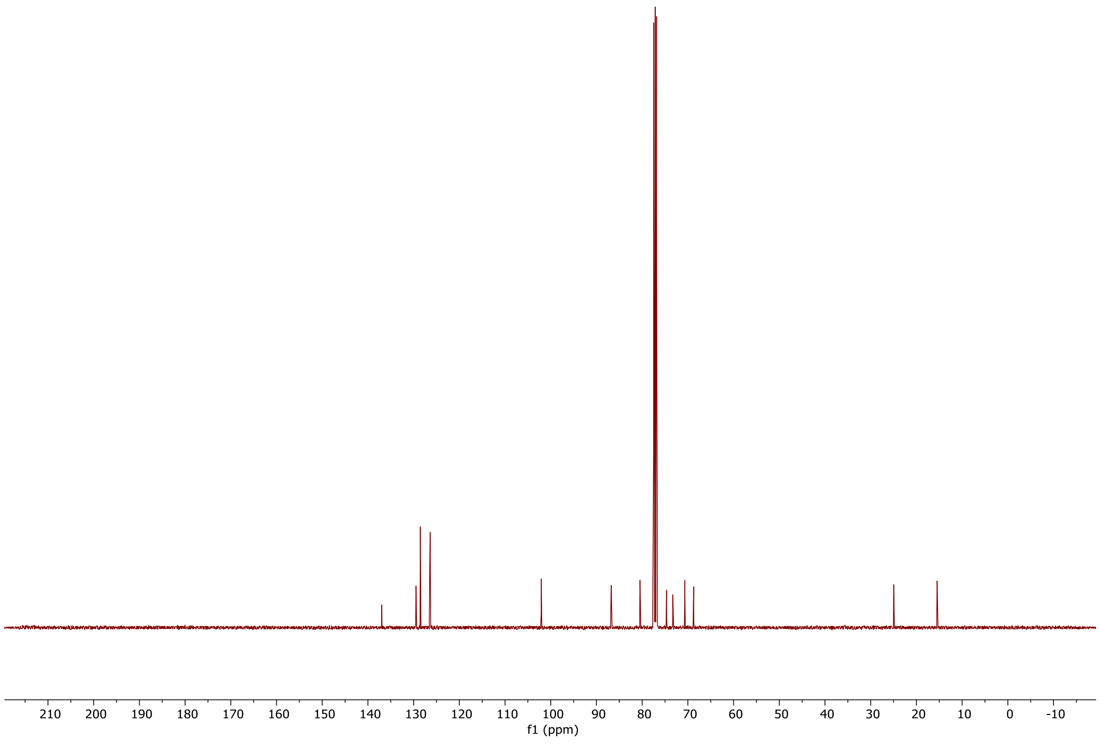

HSQC NMR of 4 ($CDCl_3$)

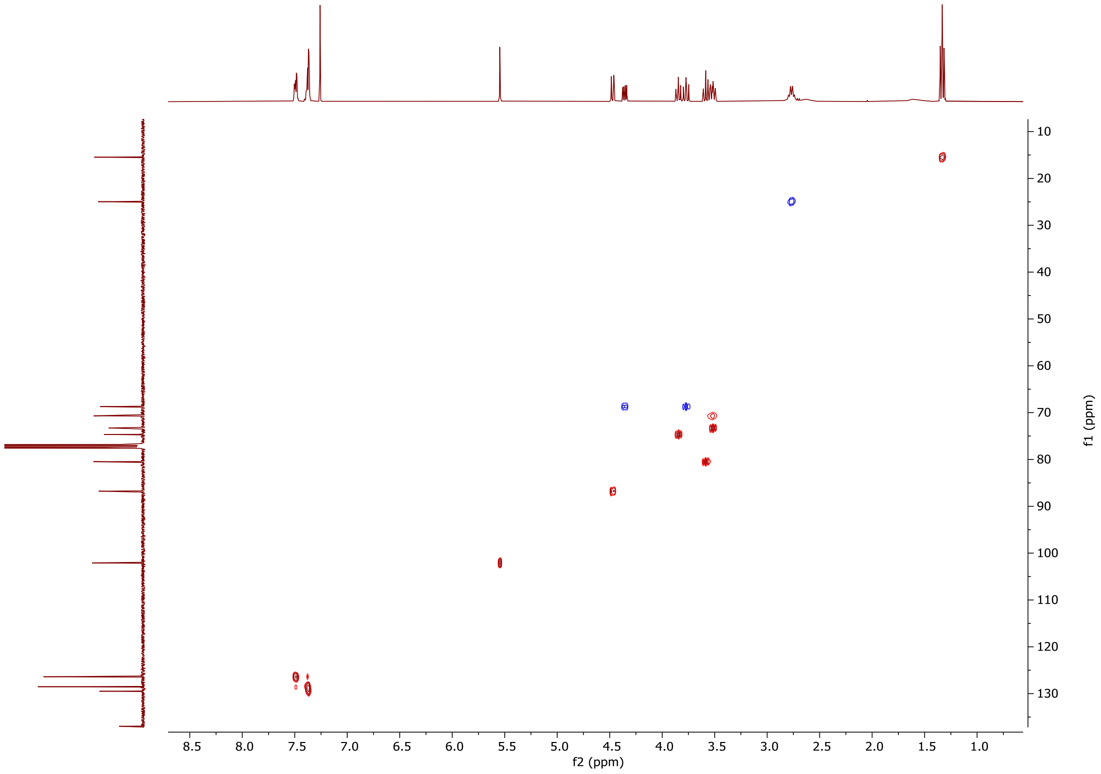

COSY NMR of 4 ($CDCl_3$)

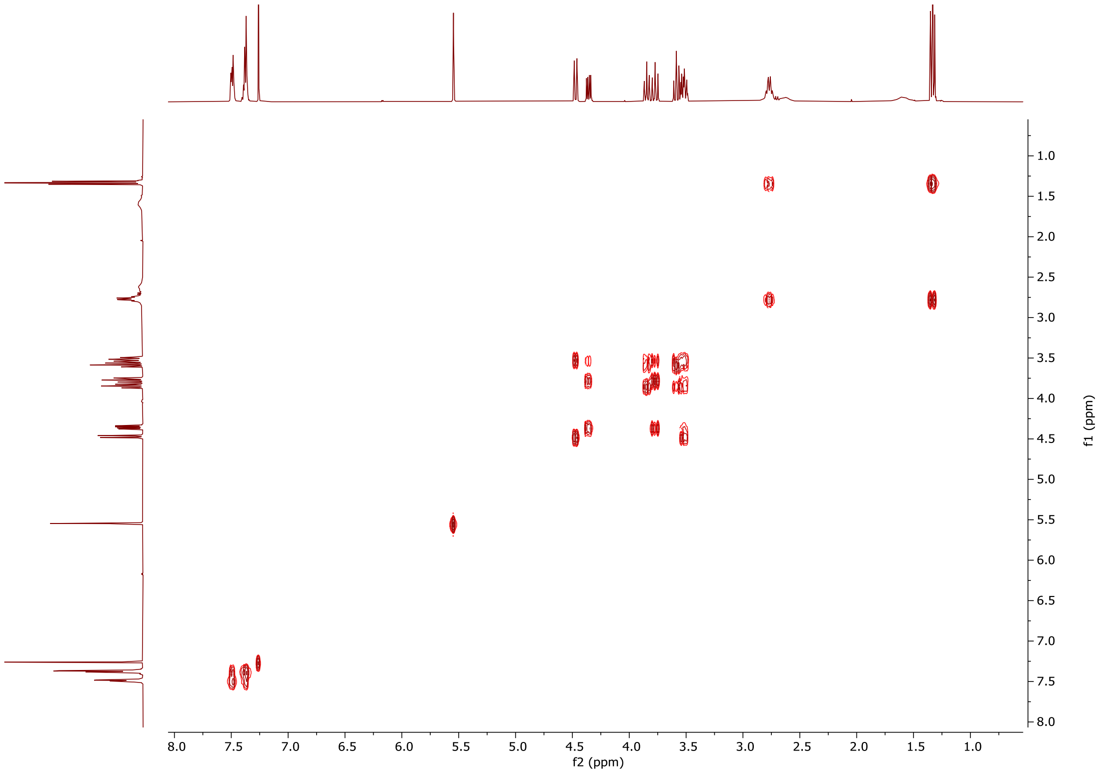

Synthesis of ethyl 2,3-di-O-benzoyl-4,6-O-benzylidene-1-thio-β-L-glucopyranoside, **5**

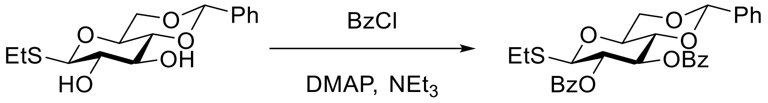

4 **5**

Ethyl 4,6-*O*-benzylidene-1-thio-β-L-glucopyranoside **4** (0.85 mg, 2.72 mmol) was dissolved in anhydrous DCM (40 mL). Triethylamine (1.1 mL, 7.9 mmol) and 4-dimethylaminopyridine (DMAP) (200 mg, 1.6 mmol) were added to the solution, while stirring. Benzoyl chloride (950 µL, 8.2 mmol) was slowly added at 0 °C and the reaction allowed to RT. Upon completion (18 h), the reaction was quenched with saturated aqueous solution of $NaHCO_3$. The mixture was washed three times with saturated aqueous solution of $NaHCO_3$ and once with brine. The organic layer was dried over Na_2SO_4 and concentrated under reduced pressure. The crude product was purified through a short plug of silica with EtOAc followed by recrystallization from hexane:EtOAc to yield **5** as a white solid (1.31 g, 93%).

^{1}H NMR (400 MHz, Chloroform-*d*) δ 8.01 – 7.89 (m, 4H), 7.56 – 7.45 (m, 2H), 7.45 – 7.28 (m, 9H), 5.81 (t, *J* = 9.4 Hz, 1H), 5.55 (s, 1H), 5.52 (dd, *J* = 10.0, 9.2 Hz, 1H), 4.82 (d, *J* = 10.0 Hz, 1H), 4.45 (dd, *J* = 10.4, 4.8 Hz, 1H), 3.93 (t, *J* = 9.5 Hz, 1H), 3.87 (t, *J* = 10.2 Hz, 1H), 3.75 (td, *J* = 9.7, 4.8 Hz, 1H), 2.77 (qd, *J* = 7.5, 4.5 Hz, 2H), 1.26 (t, *J* = 7.5 Hz, 3H). ^{13}C NMR (101 MHz, Chloroform-*d*) δ 165.73, 165.46, 136.84, 133.46, 133.27, 130.02, 129.93, 129.48, 129.26, 129.20, 128.53, 128.45, 128.35, 126.24, 101.60, 84.63, 78.91, 73.30, 71.15, 71.09, 68.72, 24.58, 14.98. $[\alpha]_D^{20}$ -16.05 (c 0.54 g/100 mL, $CHCl_3$). IR v = 2926, 1719, 1258, 1094, 1069, 709 cm^{-1}. (ESI-HRMS) m/z 543.145 $[M+Na]^+$ ($C_{29}H_{28}O_7SNa$ requires 543.145).

^{1}H NMR of 5 (400 MHz, $CDCl_3$)

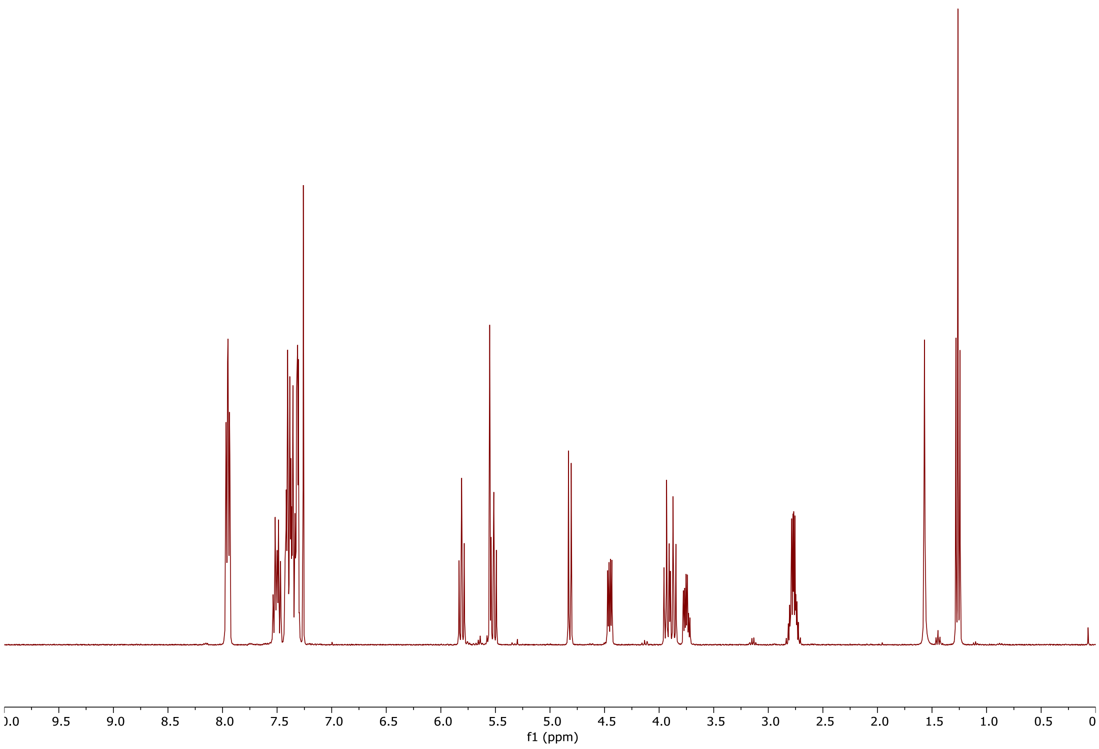

^{13}C NMR of 5 (101 MHz, $CDCl_3$)

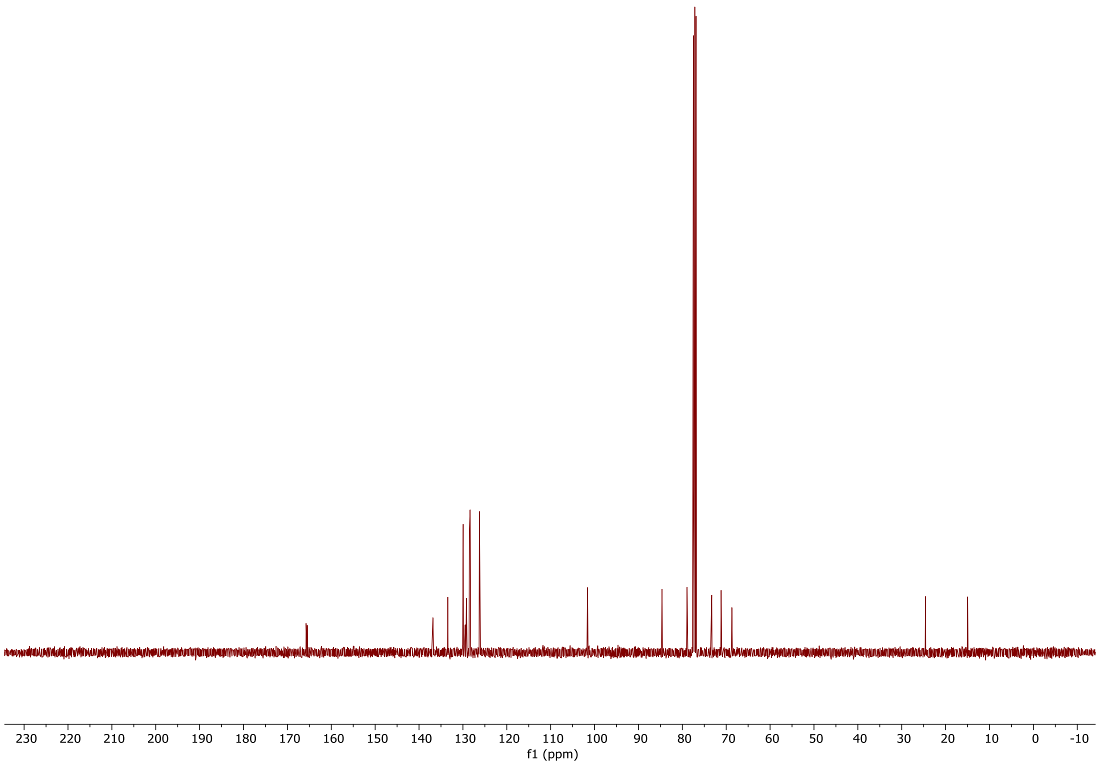

HSQC NMR of 5 ($CDCl_3$)

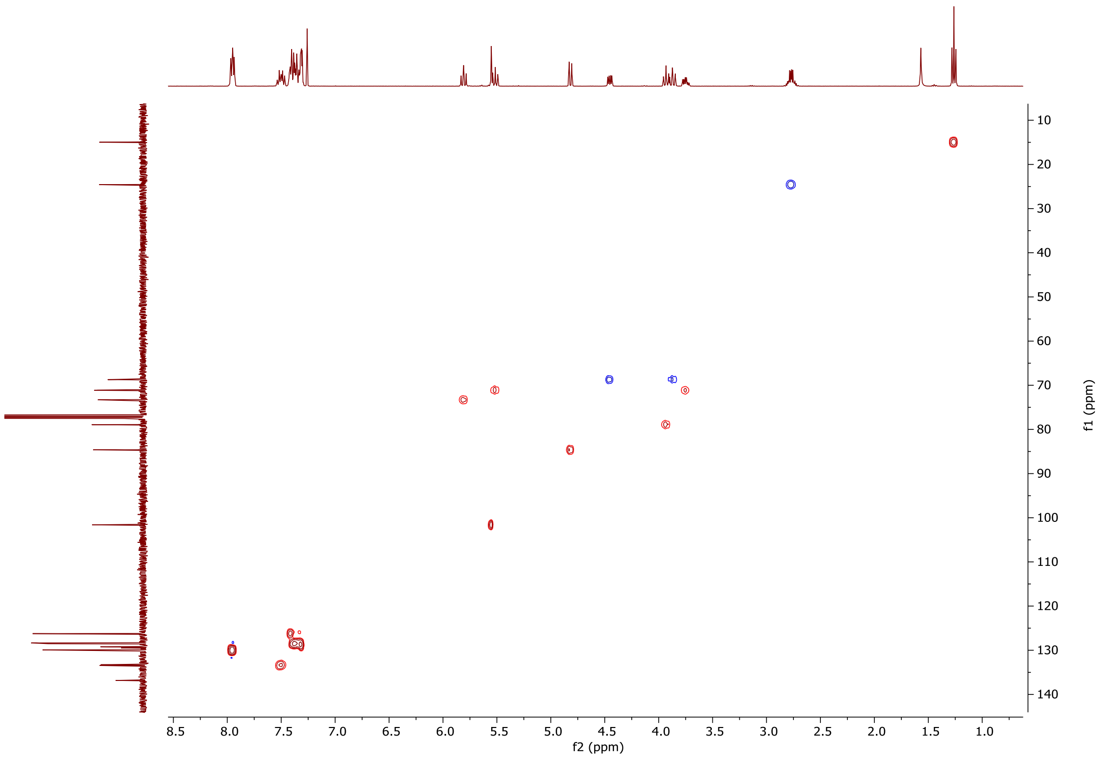

COSY NMR of 5 ($CDCl_3$)

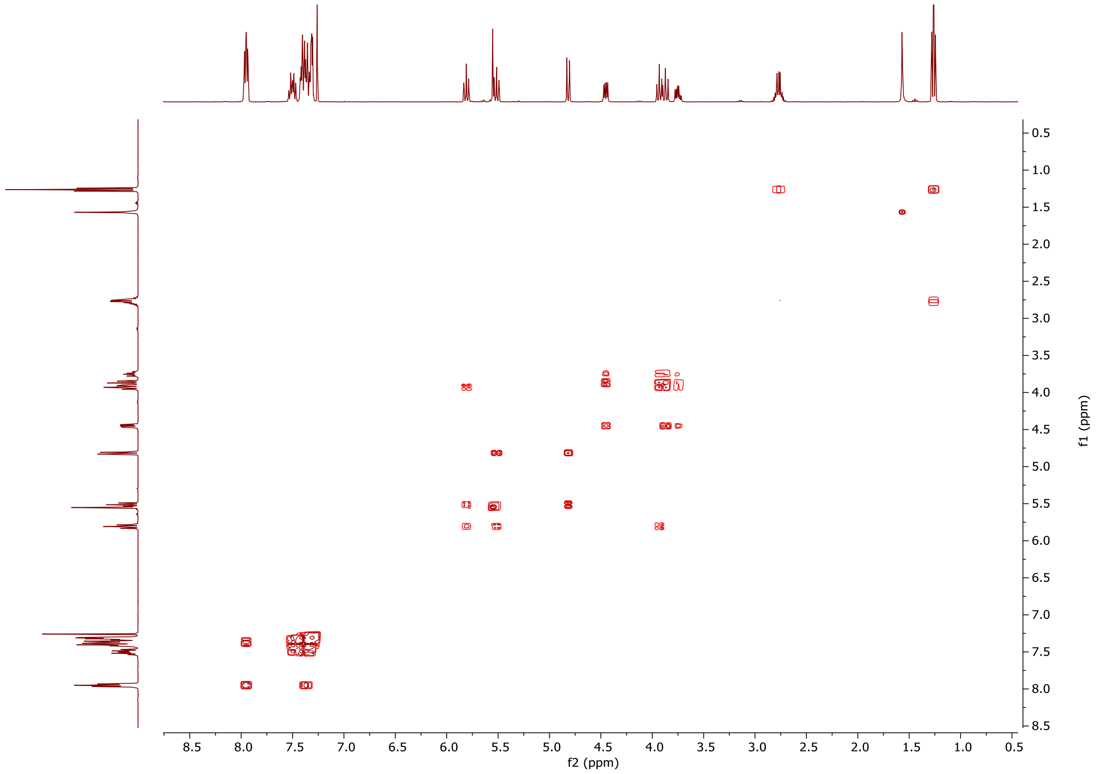

Synthesis of ethyl 2,3-di-O-benzoyl-4-O-benzyl-1-thio-β-L-glucopyranoside, **6**

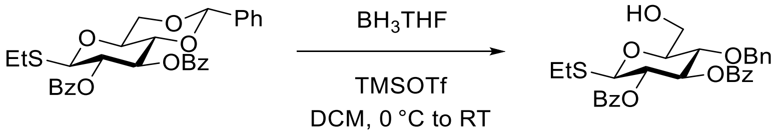

Ethyl 2,3-di-*O*-benzoyl-4,6-*O*-benzylidene-1-thio-β-L-glucopyranoside **5** (1.31 g, 2.52 mmol) was dissolved in DCM (30 mL). BH_3THF (10 mL, 10 mmol) and TMSOTf (230 μL, 0.5 mmol) were sequentially added to the stirred solution at 0 °C. The reaction was allowed to RT and quenched with saturated aqueous solution of $NaHCO_3$ after 4 h. The organic layer was washed three times with saturated aqueous solution of $NaHCO_3$ and once with brine, dried with Na_2SO_4 and concentrated in vacuum. The crude product was purified by flash column chromatography with silica (hexane:EtOAc = 3:1→3:2) to give **6** as a colorless oil (1.29 g, 98%).

^{1}H NMR (400 MHz, Chloroform-*d*) δ 7.99 – 7.86 (m, 4H), 7.56 – 7.45 (m, 2H), 7.42 – 7.29 (m, 4H), 7.22 – 7.10 (m, 5H), 5.75 (t, *J* = 9.4 Hz, 1H), 5.36 (t, *J* = 9.8 Hz, 1H), 4.74 (d, *J* = 10.0 Hz, 1H), 4.60 (s, 2H), 3.98 (dd, *J* = 12.3, 2.5 Hz, 1H), 3.93 (t, *J* = 9.5 Hz, 1H), 3.81 (dd, *J* = 12.3, 4.0 Hz, 1H), 3.62 (ddd, *J* = 9.7, 4.1, 2.5 Hz, 1H), 2.74 (qd, *J* = 7.5, 1.9 Hz, 2H), 1.25 (t, *J* = 7.5 Hz, 3H). ^{13}C NMR (101 MHz, Chloroform-*d*) δ 165.81, 165.55, 137.22, 133.37, 133.36, 129.99, 129.85, 129.46, 129.31, 128.48, 128.37, 128.15, 83.95, 79.77, 76.32, 75.52, 74.98, 70.94, 61.84, 24.67, 15.02. $[\alpha]_D^{20}$ -54.95 (c 0.89 g/100 mL, $CHCl_3$). IR *v* = 1726, 1273, 1090, 1070, 709 cm^{-1}. (ESI-HRMS) m/z 545.161 $[M+Na]^+$ ($C_{29}H_{30}O_7SNa$ requires 545.160).

^{1}H NMR of 6 (400 MHz, $CDCl_3$)

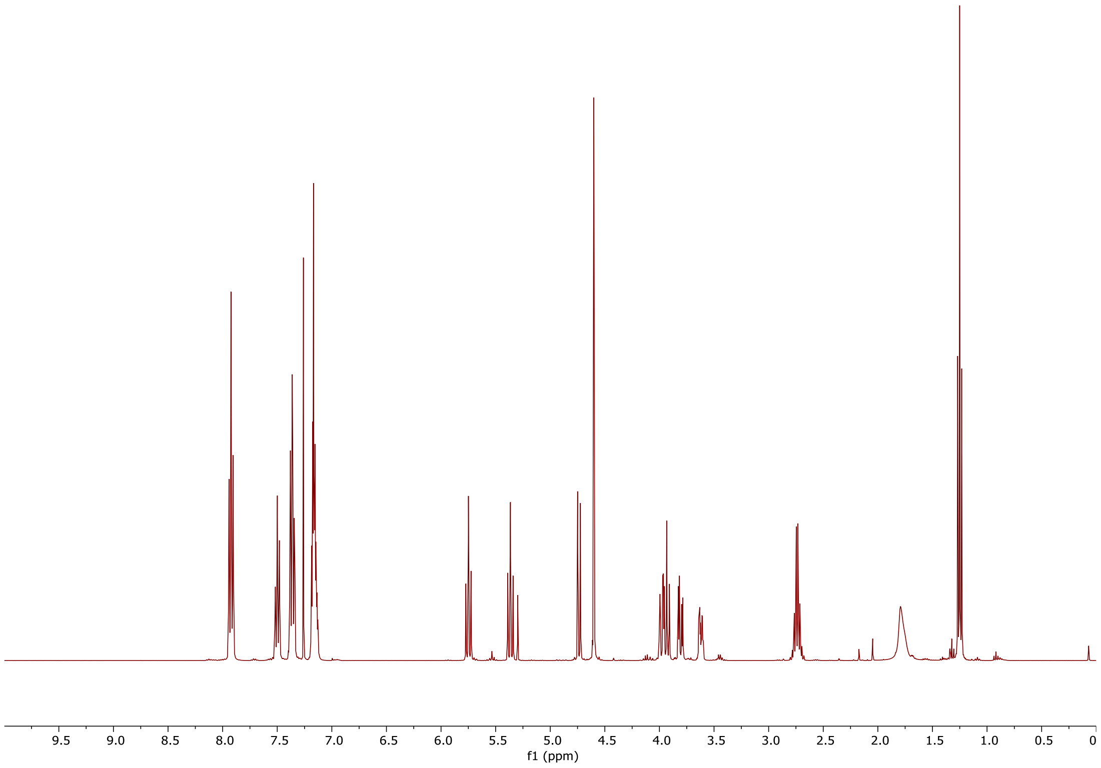

^{13}C NMR of 6 (101 MHz, $CDCl_3$)

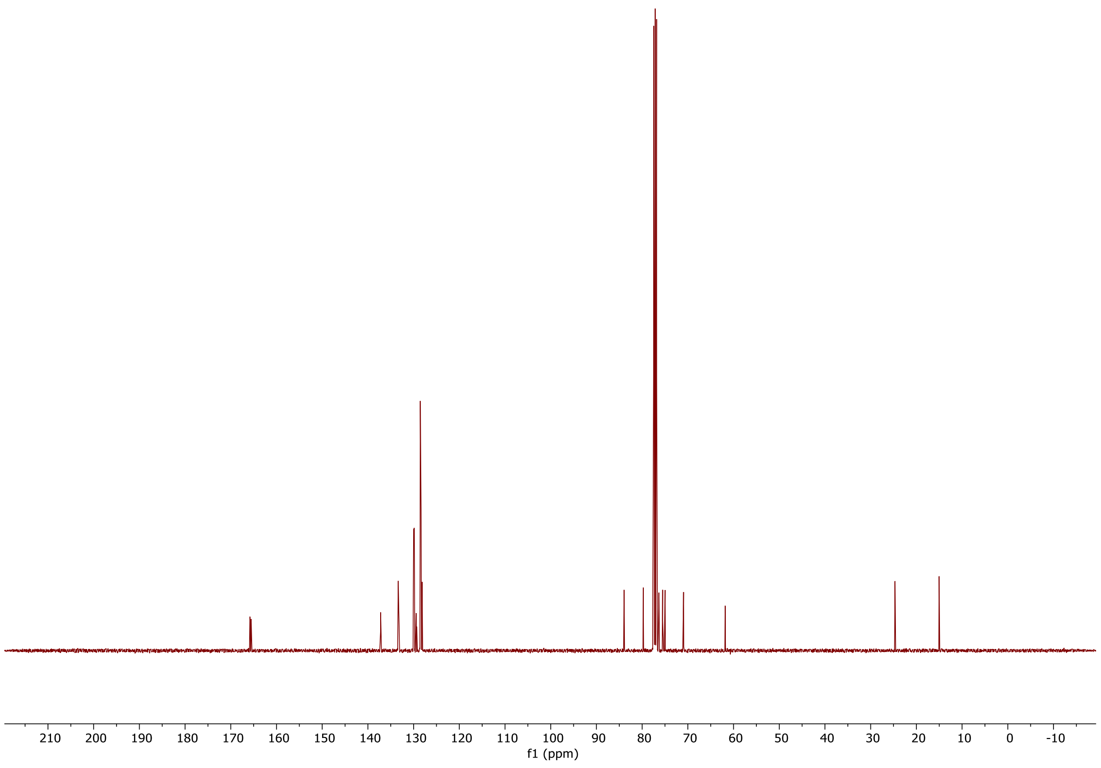

HSQC NMR of 6 ($CDCl_3$)

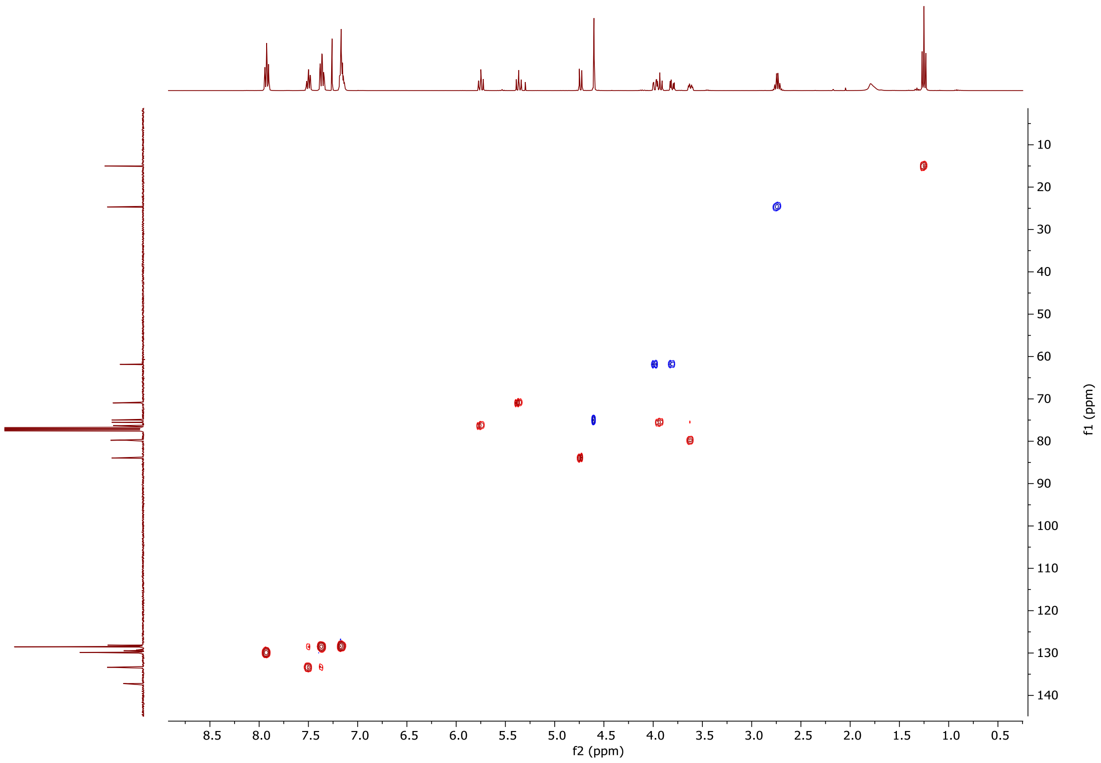

COSY NMR of 6 ($CDCl_3$)

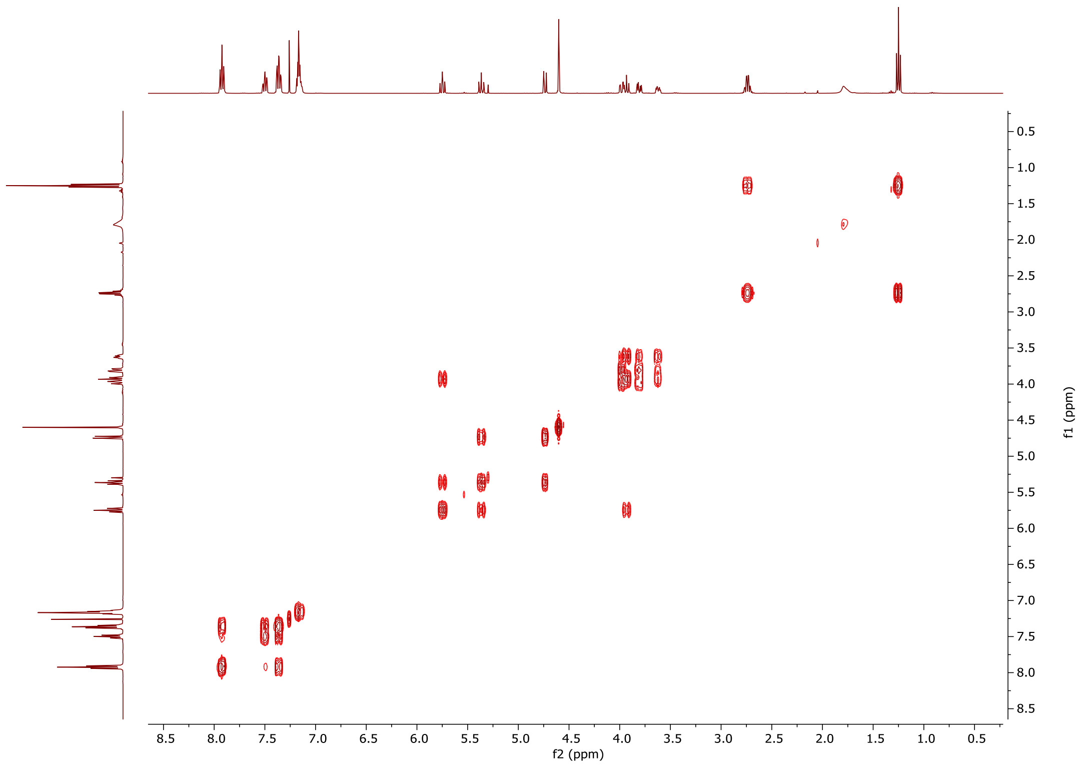

Synthesis of ethyl 2,3-di-O-benzoyl-4-O-benzyl-6-O-(9-fluorenylmethoxycarbonyl)-1-thio-β-L-glucopyranoside, **7**

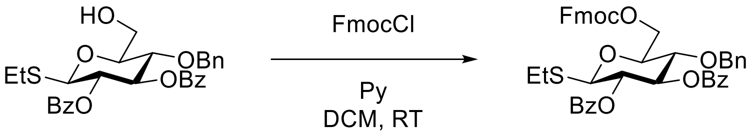

6 **7**

Ethyl 2,3-di-*O*-benzoyl-4-*O*-benzyl-1-thio-β-L-glucopyranoside **6** (1.29 g, 2.46 mmol) was dissolved in DCM (30 mL) and pyridine was added (600 µL, 7.43 mmol). FmocCl (1.28 g, 4.95 mmol) was dissolved in DCM (5 mL) and added to the reaction mixture under Ar atmosphere. The yellow solution was stirred for 4 h then quenched with a 1 M solution of HCl. The organic layer was washed three times with 1 M HCl and once with brine. The crude compound was purified with flash column chromatography (toluene:DCM = 4:1→3:1 then toluene:EtOAc = 4:1) to give **7** as a white solid (1.60 g, 87%).

^{1}H NMR (400 MHz, Chloroform-*d*) δ 8.00 – 7.89 (m, 4H), 7.78 (d, *J* = 7.5 Hz, 2H), 7.70 – 7.59 (m, 2H), 7.51 (td, *J* = 7.3, 5.5 Hz, 2H), 7.46 – 7.31 (m, 8H), 7.21 – 7.10 (m, 5H), 5.77 (t, *J* = 9.3 Hz, 1H), 5.43 (t, *J* = 9.8 Hz, 1H), 4.72 (d, *J* = 10.0 Hz, 1H), 4.64 – 4.51 (m, 3H), 4.50 – 4.33 (m, 3H), 4.29 (t, *J* = 7.4 Hz, 1H), 3.92 (t, *J* = 9.4 Hz, 1H), 3.81 (ddd, *J* = 9.9, 4.9, 2.1 Hz, 1H), 2.83 – 2.65 (m, 2H), 1.25 (t, *J* = 7.4 Hz, 3H). ^{13}C NMR (101 MHz, Chloroform-*d*) δ 165.77, 165.53, 155.04, 143.50, 143.39, 141.45, 136.91, 133.44, 133.37, 130.01, 129.88, 129.40, 129.34, 128.59, 128.58, 128.48, 128.43, 128.25, 128.08, 127.36, 127.34, 125.36, 125.30, 120.24, 83.86, 77.48, 77.26, 76.43, 75.77, 75.00, 70.78, 70.22, 66.45, 46.85, 24.52, 15.06. $[\alpha]_D^{20}$ -44.69 (c 1.03 g/100 mL, $CHCl_3$). IR *v* = 1727, 1251, 1088, 1069, 741, 708 cm^{-1}. (ESI-HRMS) m/z 767.232 $[M+Na]^+$ ($C_{44}H_{40}O_9SNa$ requires 767.229).

^{1}H NMR of 7 (400 MHz, $CDCl_3$)

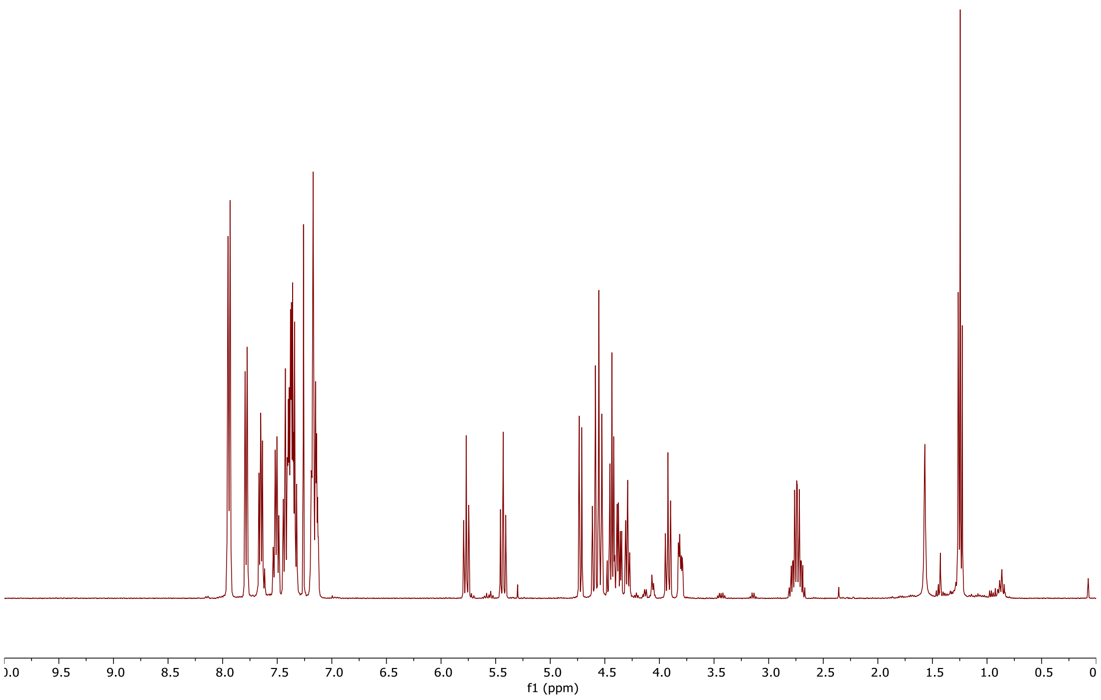

^{13}C NMR of 7 (101 MHz, $CDCl_3$)

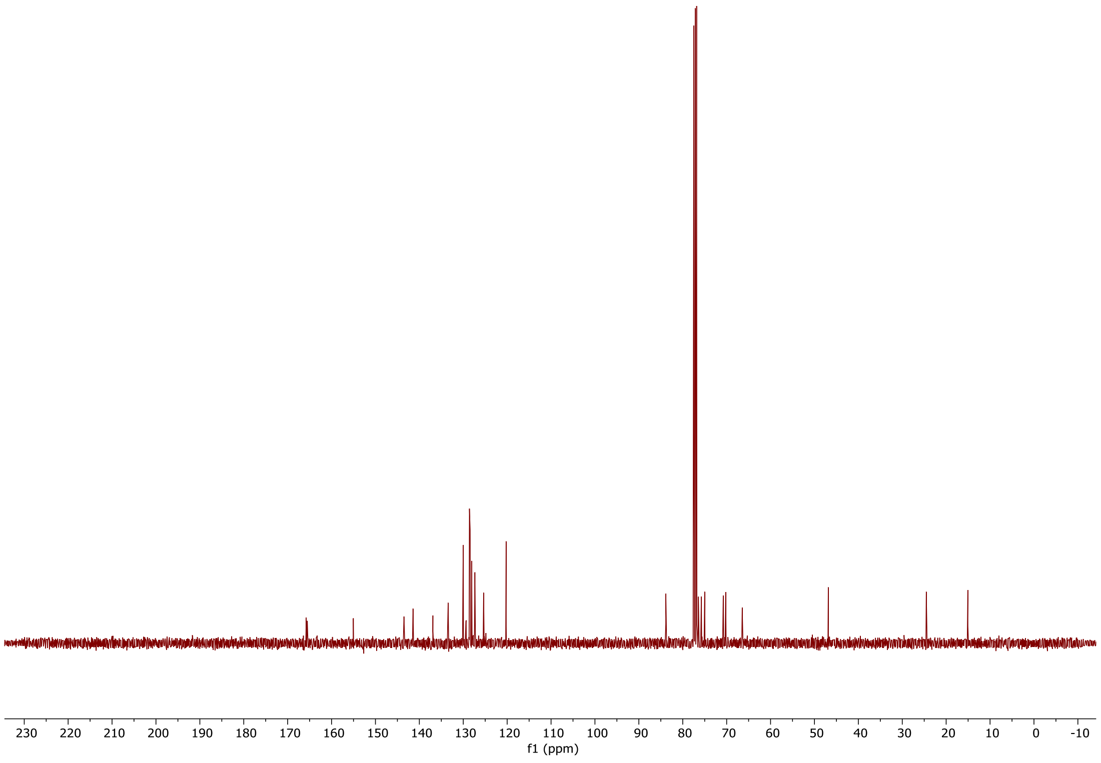

HSQC NMR of 7 ($CDCl_3$)

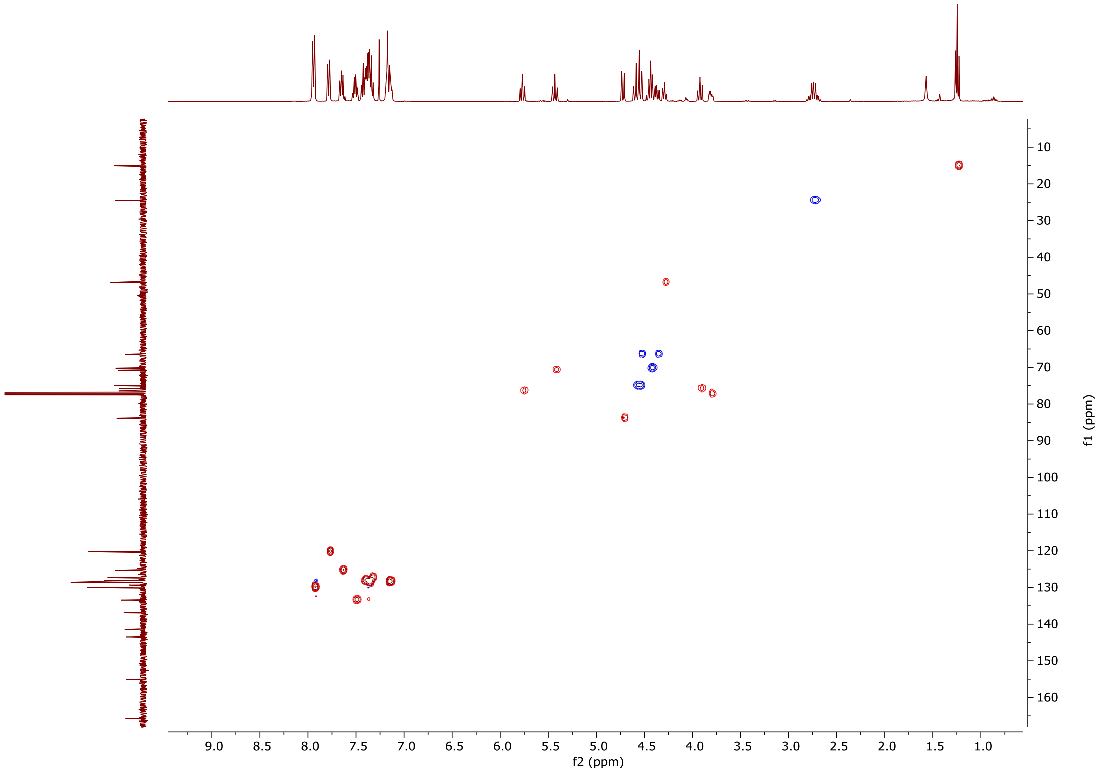

COSY NMR of 7 ($CDCl_3$)

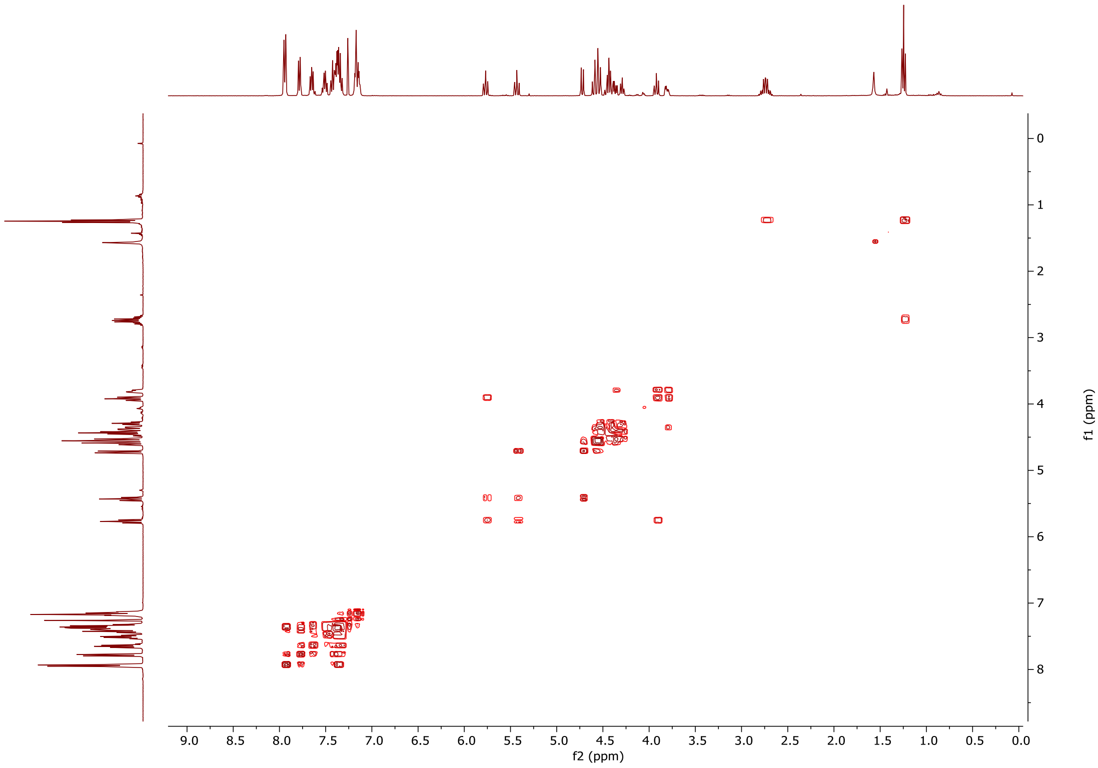

3.2. Synthesis of LL

Synthesis of benzyl 2,3-O-dibenzoyl-4-O-benzyl-β-L-glucopyranoside, **8**

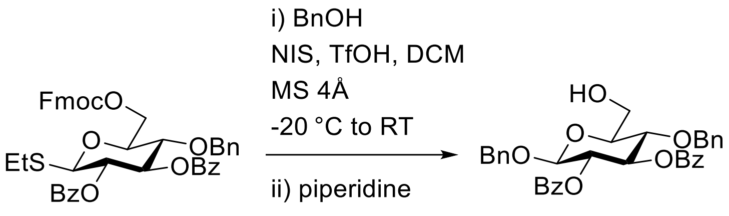

7 **8**

Ethyl 2,3-di-*O*-benzoyl-4-*O*-benzyl-6-*O*-(9-fluorenylmethoxycarbonyl)-1-thio-*β*-*L*-glucopyranoside **7** (60 mg, 0.08 mmol) and benzyl alcohol (17 µL, 0.16 mmol) were dissolved in anhydrous DCM (800 µL). The solution was then stirred with molecular sieves (4 Å) for 1 h at RT under Ar atmosphere and then cooled to -20 °C. The activator solution (300 µL of a 200 mg/mL solution of NIS in DCM:dioxane 2:1 with 1% TfOH) was added dropwise and the reaction was stirred for 5 min at -20 °C, after which time the cooling bath was removed and the reaction allowed to RT. After 1 h, ESI-MS indicated the disappearance of **7**. Piperidine (0.5 mL) was added and the reaction was stirred at RT for an additional 1h. The reaction was diluted with DCM and washed once with 1 M HCl, once with saturated aqueous solution of $NaHCO_3$, and once with brine. The crude compound was purified by column chromatography (hexane:EtOAc = 3:1→2:1→1:1) to give **8** as a white solid (42 mg, 91%).

^{1}H NMR (600 MHz, Chloroform-*d*) δ 7.92 (ddt, J = 12.7, 7.0, 1.5 Hz, 3H), 7.55 – 7.47 (m, 2H), 7.41 – 7.33 (m, 4H), 7.25 – 7.11 (m, 9H), 5.68 (t, J = 9.6 Hz, 1H), 5.45 – 5.38 (m, 1H), 4.87 (d, J = 12.6 Hz, 1H), 4.76 (d, J = 7.9 Hz, 1H), 4.68 (dd, J = 12.7, 1.7 Hz, 1H), 4.59 (s, 2H), 4.00 – 3.91 (m, 2H), 3.81 (dd, J = 12.1, 4.0 Hz, 1H), 3.57 (ddd, J = 9.7, 4.0, 2.5 Hz, 1H). ^{13}C NMR (151 MHz, Chloroform-*d*) δ 165.83, 165.47, 137.33, 136.95, 133.33, 133.27, 130.02, 129.91, 129.56, 128.53, 128.34, 128.13, 128.05, 127.84, 99.86, 75.73, 75.60, 75.12, 74.95, 72.19, 71.13, 61.78. $[\alpha]_D^{20}$ -31.39 (c 1.07 g/100 mL, $CHCl_3$). IR *v* = 3447, 2927, 1725, 1272, 1094, 1069, 1027, 708, 699 cm^{-1}. (ESI-HRMS) m/z 591.200 [M+Na]$^+$ ($C_{34}H_{32}O_8Na$ requires 591.199).

^{1}H NMR of 8 (600 MHz, $CDCl_3$)

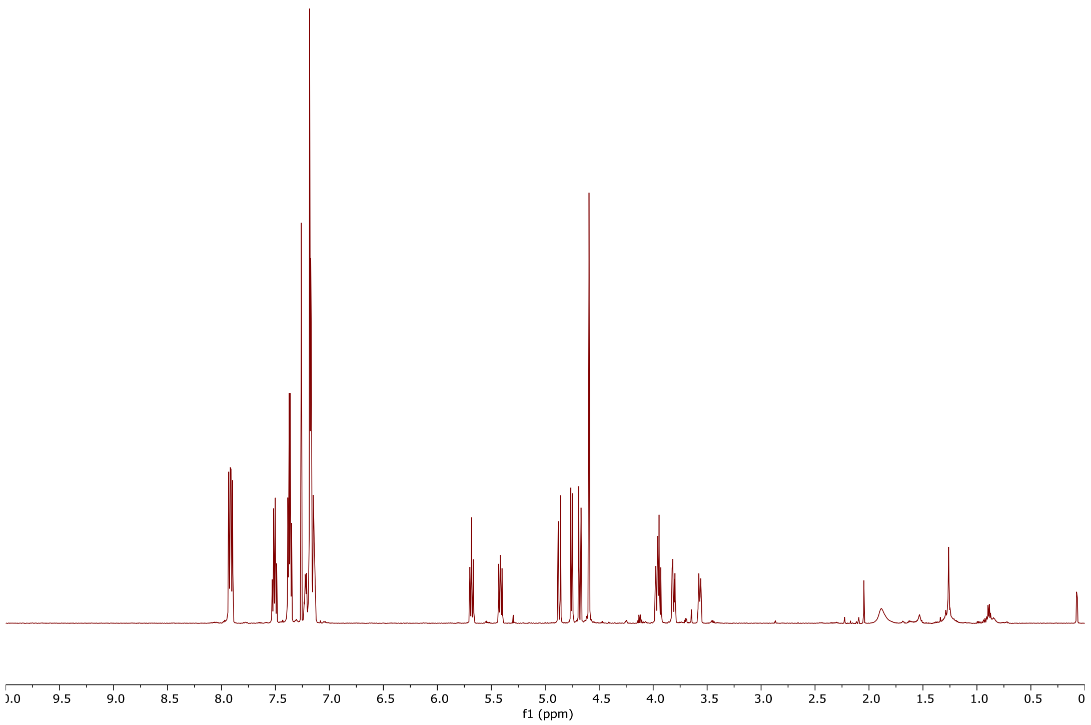

^{13}C NMR of 8 (151 MHz, $CDCl_3$)

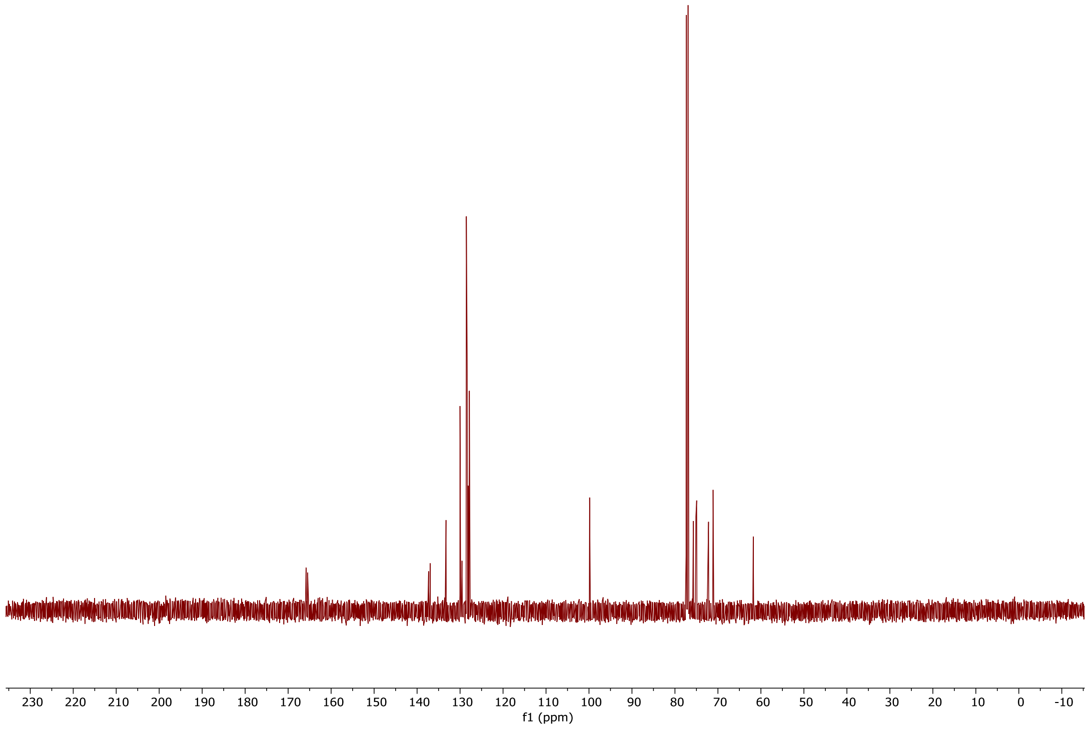

HSQC NMR of 8 ($CDCl_3$)

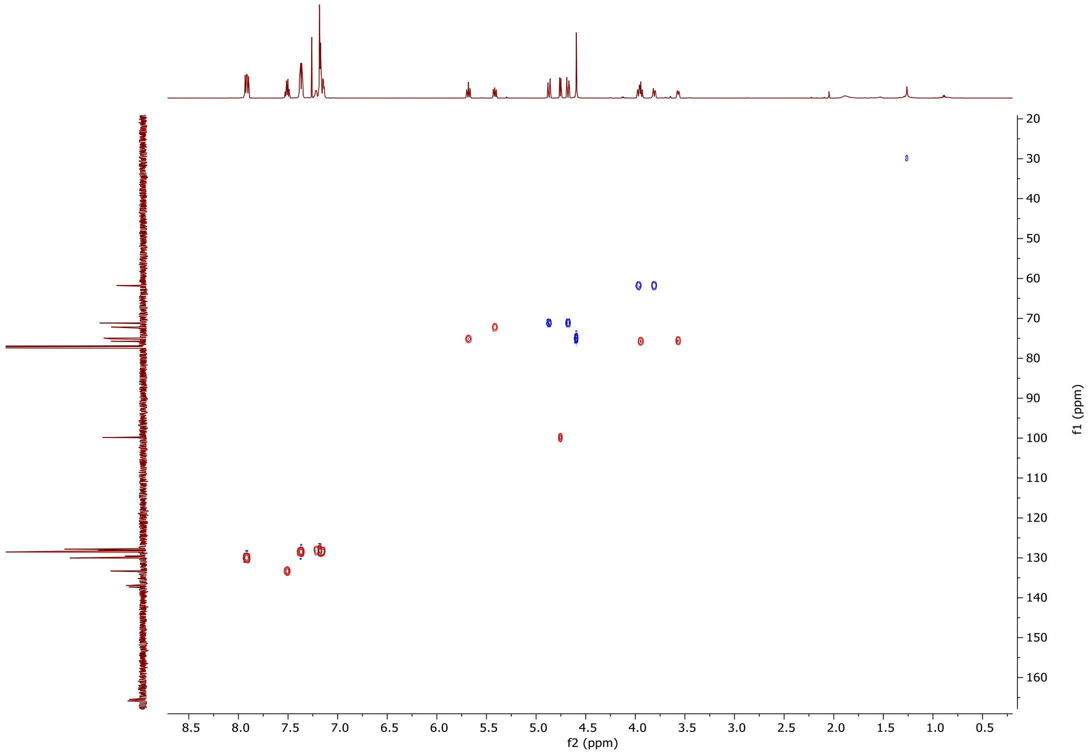

COSY NMR of 8 ($CDCl_3$)

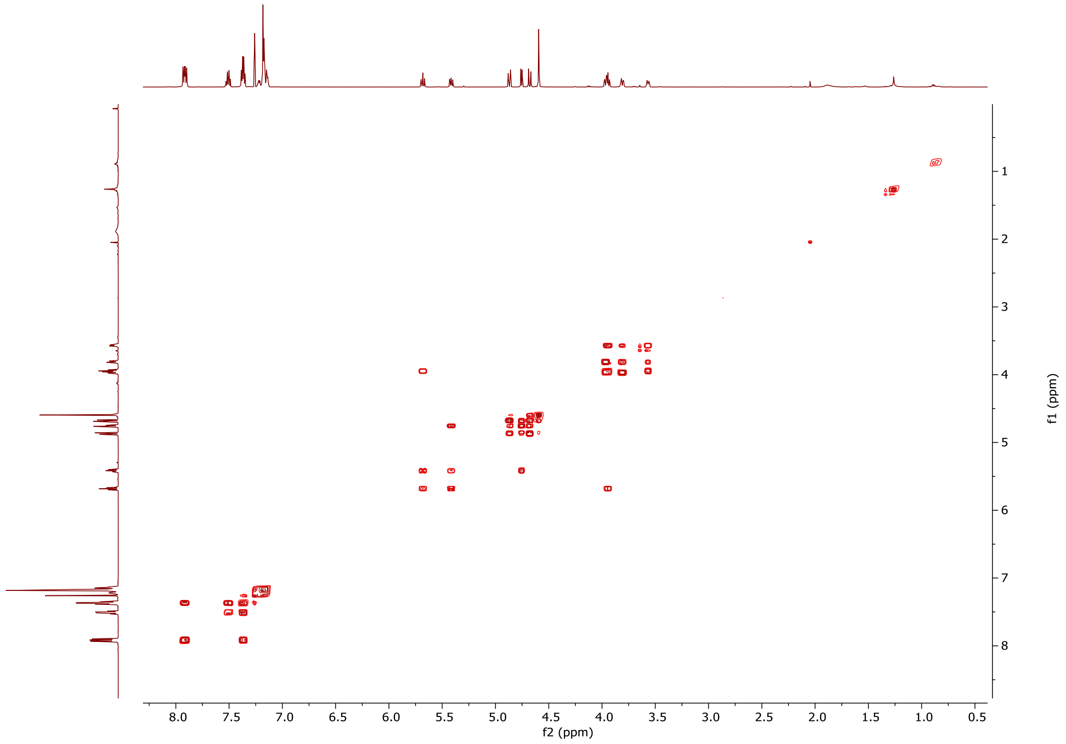

Synthesis of **LL**

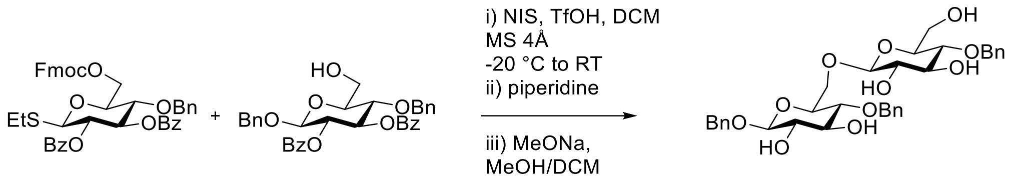

7 **8** **LL**

Ethyl 2,3-di-*O*-benzoyl-4-*O*-benzyl-6-*O*-(9-fluorenylmethoxycarbonyl)-1-thio-β-*L*-glucopyranoside **7** (69 mg, 0.093 mmol) and benzyl 2,3-*O*-dibenzoyl-4-*O*-benzyl-β-*L*-glucopyranoside **8** (42 mg, 0.074 mmol) were dissolved in anhydrous DCM (1 mL). The solution was stirred for 1 h with molecular sieves (4 Å) at RT under Ar atmosphere and then cooled to -20 °C. The activator solution (200 µL of a 200 mg/mL solution of NIS in DCM:dioxane 2:1 with 1% TfOH) was added dropwise and the reaction was stirred for 5 min at -20 °C, after which time the cooling bath was removed and the reaction allowed to RT. After 1 h, ESI-MS indicated the disappearance of **8**. Piperidine (0.5 mL) was added and the reaction was stirred at RT for an additional 1 h. The reaction was diluted with DCM and washed once with 1 M HCl, once with saturated aqueous solution of $NaHCO_3$ and once with brine. The crude compound was purified by column chromatography (hexane:EtOAc = 3:1→2:1→1:1→2:3). The fully protected compound (52 mg, 0.051 mmol) was dissolved in MeOH:DCM (4 mL, 1:1). MeONa in MeOH (0.5 M, 3 equiv. per benzoyl ester) was added and the solution was stirred at room temperature for 16 h, neutralized with Amberlite IR-120 (H^+ form) resin, filtered and concentrated *in vacuo*. The resulting yellow oil was purified by column chromatography (DCM:MeOH = 15:1) and recrystallized from DCM:hexane to give **LL** as white solid (31 mg, 75% over 3 steps).

^{1}H NMR (400 MHz, Methanol-d_4) δ 7.51 – 7.16 (m, 15H), 5.01 – 4.89 (m, 3H), 4.75 – 4.61 (m, 3H), 4.38 (d, J = 7.8 Hz, 1H), 4.33 (d, J = 7.8 Hz, 1H), 4.15 (d, J = 10.9 Hz, 1H), 3.88 – 3.72 (m, 2H), 3.72 – 3.62 (m, 1H), 3.62 – 3.45 (m, 4H), 3.41 (t, J = 9.3 Hz, 1H), 3.36 – 3.18 (m, 3H). ^{13}C NMR (101 MHz, Methanol-d_4) δ 140.10, 140.04, 139.10, 129.31, 129.28, 129.16, 129.13, 129.08, 128.69, 128.65, 105.00, 103.42, 79.34, 79.12, 78.51, 78.37, 77.05, 76.17, 75.73, 75.67, 75.37, 75.31, 71.97, 69.56, 62.32. (ESI-HRMS) m/z 635.245 $[M+Na]^+$ ($C_{33}H_{40}O_{11}Na$ requires 635.246).

^{1}H NMR of LL (400 MHz, CD_3OD)

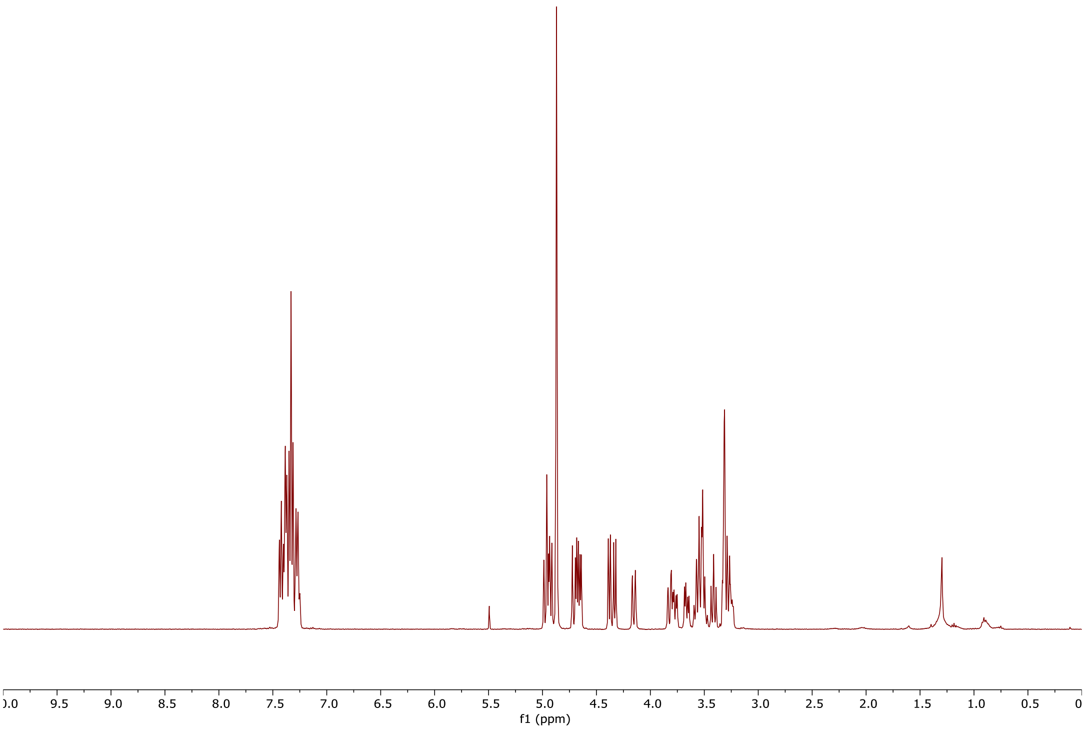

^{13}C NMR of LL (101 MHz, CD_3OD)

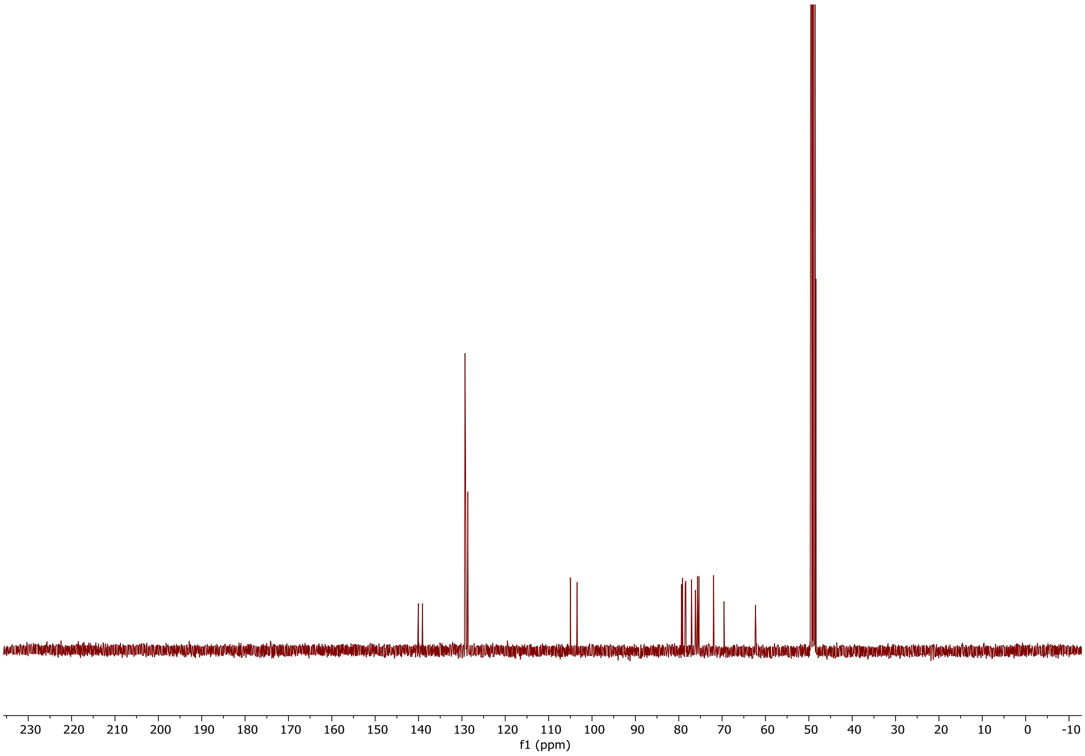

HSQC NMR of LL (400 MHz, CD_3OD)

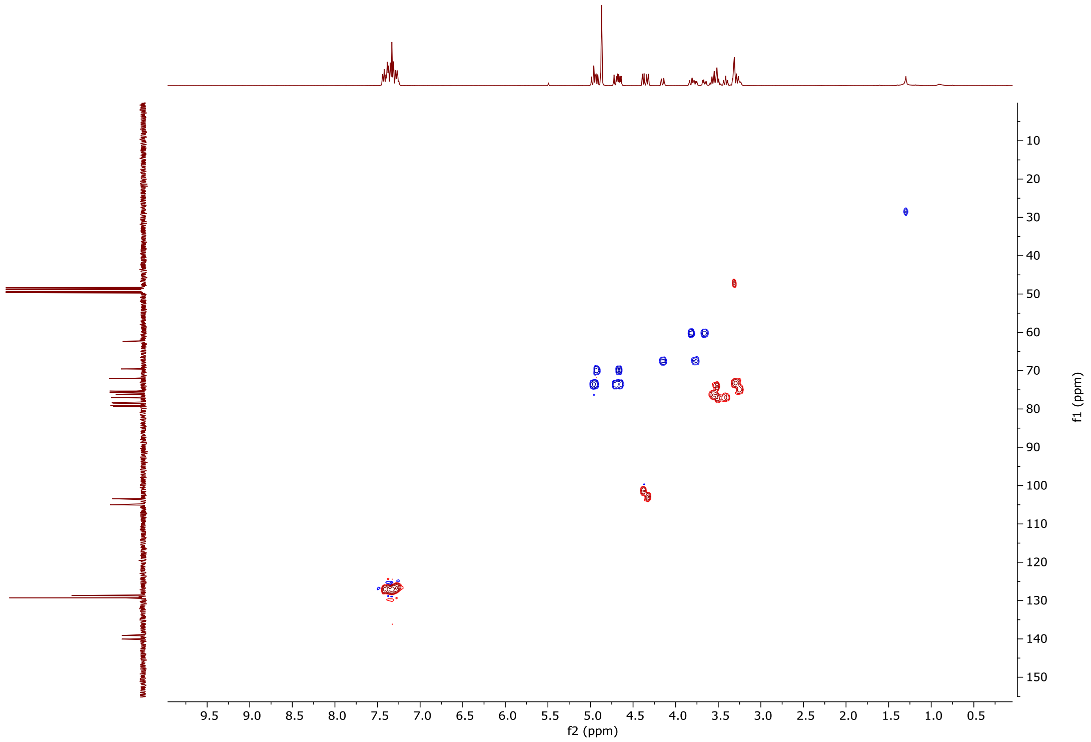

COSY NMR of LL (400 MHz, CD_3OD)

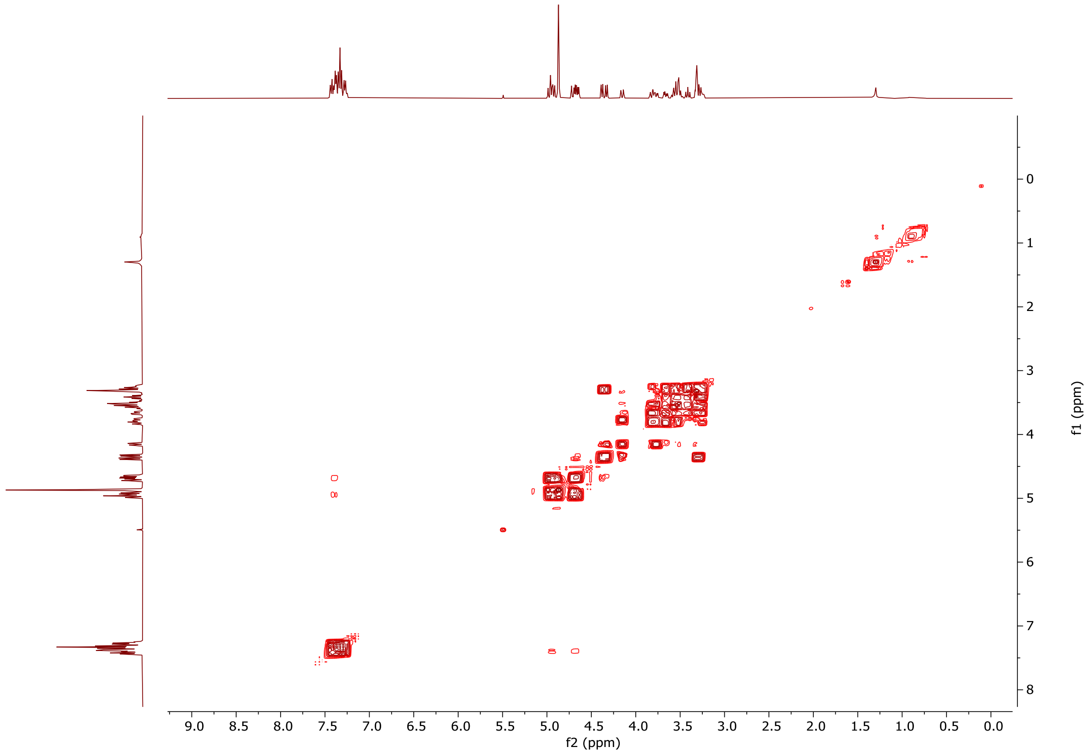

4. Self-Assembly

A) **Solvent-switch method (s):** Stock solutions of the disaccharides (20, 40 and 100 mg mL^{-1}) in HFIP were diluted with water to reach a final concentration of 2 mg mL^{-1} with different ratio of water and HFIP. The temperature was controlled with an oil bath. When not mentioned, the conditions are 2 % HFIP in water at room temperature. The samples were incubated for 3 days without agitation before the measurement.

Table S1. Summary of the samples prepared by the solvent-switch method (s).

<table>
<tr><th>Sample</th><th>Compound</th><th>HFIP content (%)</th><th>Temperature</th><th>Supramolecular morphology</th><th>Dimensions (Width/Length) (μm)</th></tr>
<tr><td rowspan="3">DD(s)</td><td rowspan="5">DD</td><td rowspan="3">2</td><td>75°C</td><td>Lamellae</td><td>0.5 ~ 10 /
100 ~ 800</td></tr>
<tr><td>R.T.</td><td rowspan="4">Left handed helical fibers</td><td>0.1 ~ 1.0 /
10 ~ 50</td></tr>
<tr><td>5°C</td><td>0.1 ~ 0.7 /
5 ~ 25</td></tr>
<tr><td>DD(s-5%)</td><td>5</td><td rowspan="8">R.T.</td><td>0.1 ~ 2.0 /
10 ~ 100</td></tr>
<tr><td>DD(s-10 %)</td><td>10</td><td>0.1 ~ 5.0 /
10 ~ 200</td></tr>
<tr><td>LL(s)</td><td rowspan="3">LL</td><td>2</td><td rowspan="3">Right handed Helical fiber</td><td>0.1 ~ 2.0 /
5 ~ 40</td></tr>
<tr><td>LL(s-5 %)</td><td>5</td><td>0.1 ~ 5.0 /
10 ~ 100</td></tr>
<tr><td>LL(s-10 %)</td><td>10</td><td>0.1 ~ 5.0 /
10 ~ 200</td></tr>
<tr><td>DD-LL(s)</td><td>DD:LL = 1:1</td><td rowspan="3">2</td><td rowspan="3">Lamellae</td><td>15 ~ 20 /
100 ~ 200</td></tr>
<tr><td></td><td>DD:LL = 1:2</td><td>0.1 ~ 20 /
2 ~ 50</td></tr>
<tr><td></td><td>DD:LL = 2:1</td><td>0.1 ~ 15 /
1 ~ 50</td></tr>
</table>

B) Film-rehydration method (fr): 2 µL of the disaccharide solution in HFIP (1, 10 and 100 mg mL^{-1}) were drop casted and dried on the substrate for 12 h. The dried film was transferred into a humidity chamber and observed at different time scale. When not mentioned, the concentration is 100 mg mL^{-1}.

Table S2. Summary of the samples prepared by the film-rehydration method (fr).

Sample	Compound	Concentration (mg mL^{-1})	Temperature
**DD(fr-1)**	DD	1	R.T.
**DD(fr-10)**		10	
**DD(fr)**		100	
**LL(fr)**	LL		
**DD-LL(fr)**	DD:LL = 1:1		

5. NMR analysis

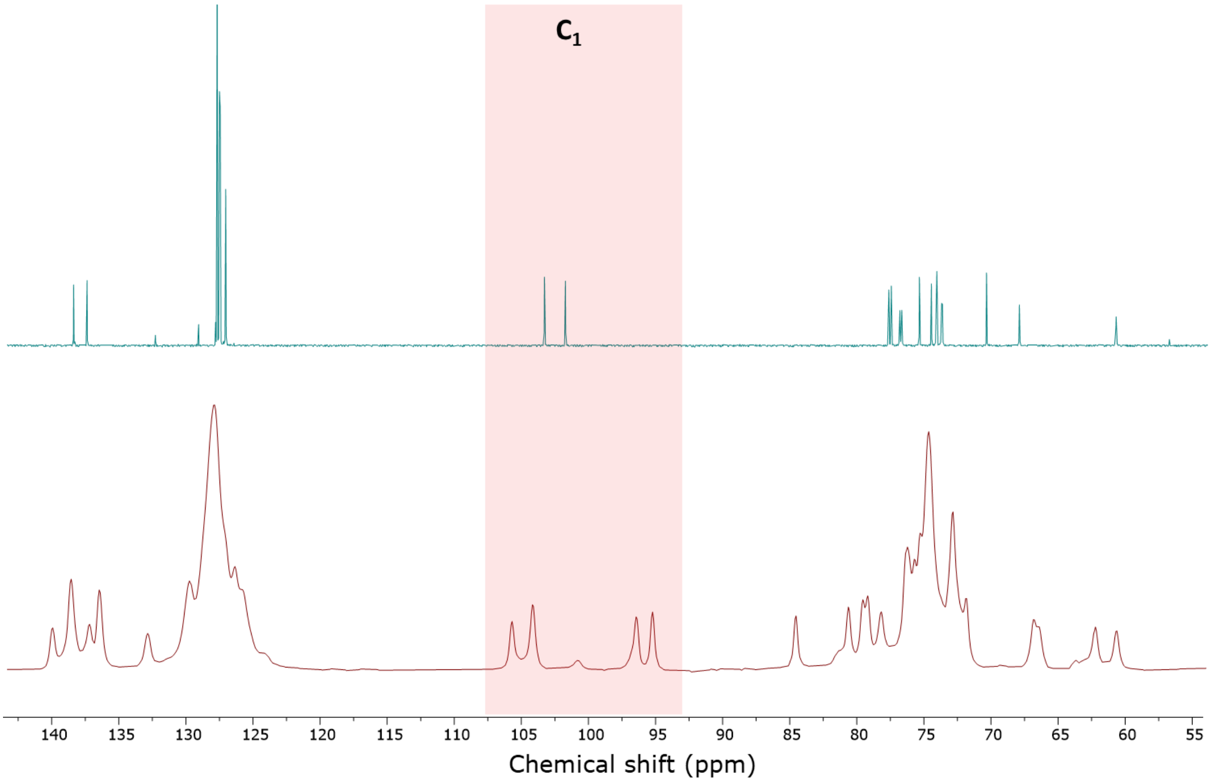

Figure S1. NMR analysis of compound **DD** in solution ($CDCl_3$, top) and in solid state (bottom). The splitting of the two anomeric carbons signals (C_1, highlighted with the red box) indicates the presence of two differently oriented sets of dimers in the unit cell.

6. MicroED analysis

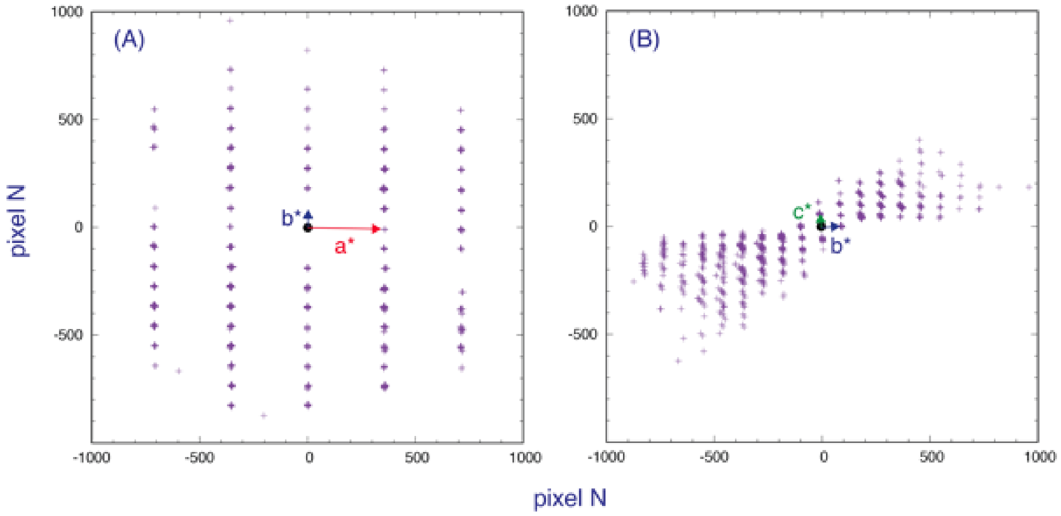

Figure S2. Reciprocal lattice reconstructed from the tilt-series microED patterns of **DD** in (A) a*b* projection and (B) b*c* projection.

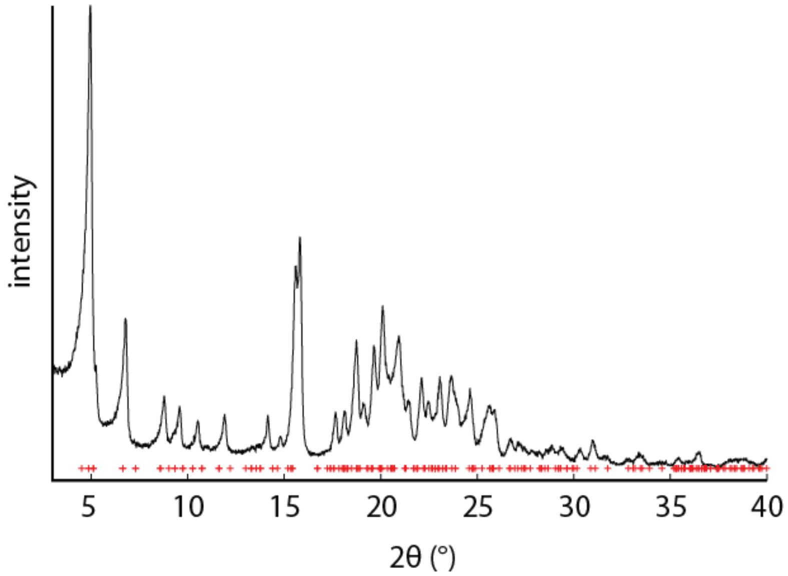

Figure S3. Powder X-ray diffraction pattern (black line) and reflection positions calculated based on the MicroED unit cell (red cross) for **DD(s)**.

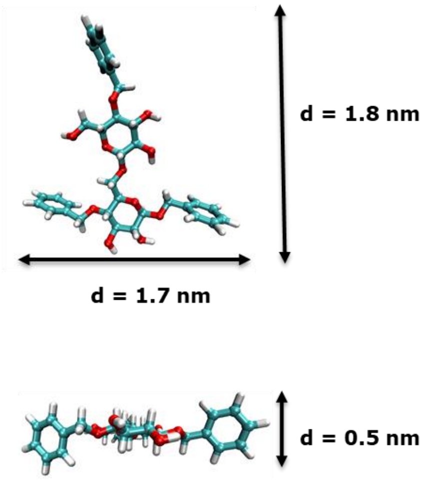

Figure S4. Energy minimized computational model of **DD**. The initial conformation of the structure was constructed with tleap. The topology was converted to gromacs format using the glycam2gmx.pl script and solvated with 2100 water molecules (TIP5P[2]) using gromacs tools.[3]

7. Morphological analysis at different T

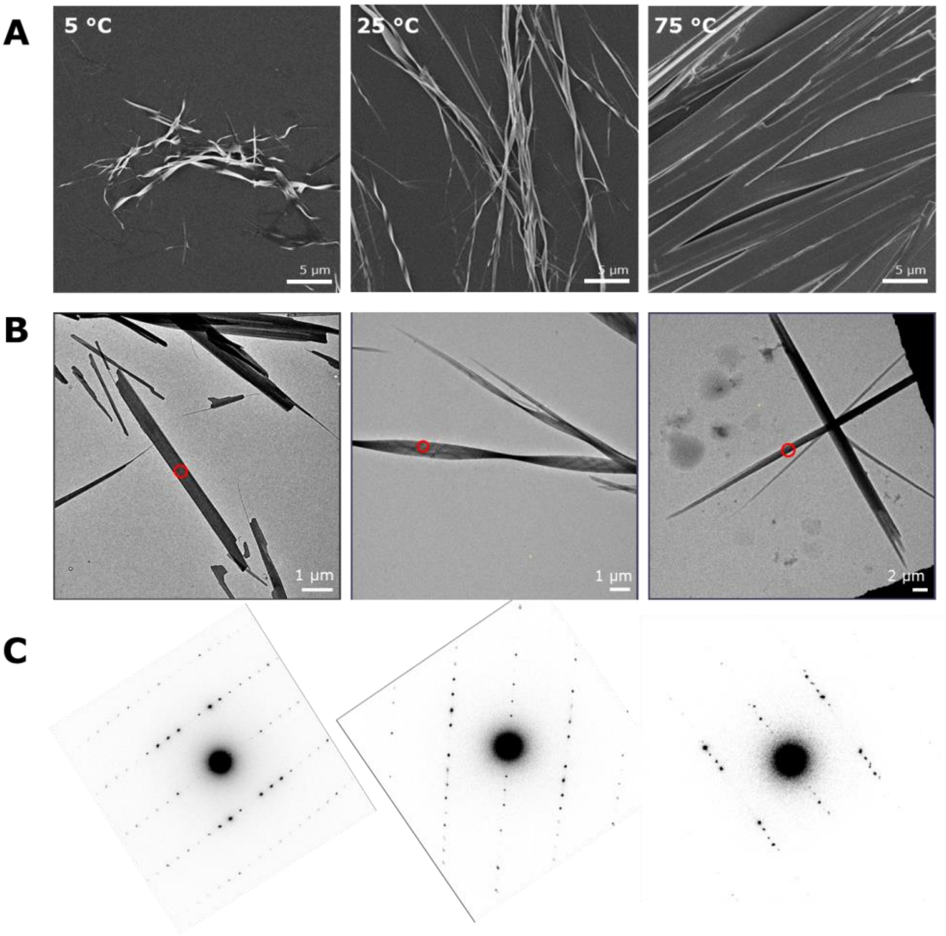

Figure S5. SEM (A) and TEM (B) images and electron diffraction patterns (C, from a red circled area in B) of **DD(s)** prepared at different temperature (left: 5 °C, middle: 25 °C, and right: 75 °C). The results indicate that the three compounds have the same crystal structures, but different fibril dimensions.

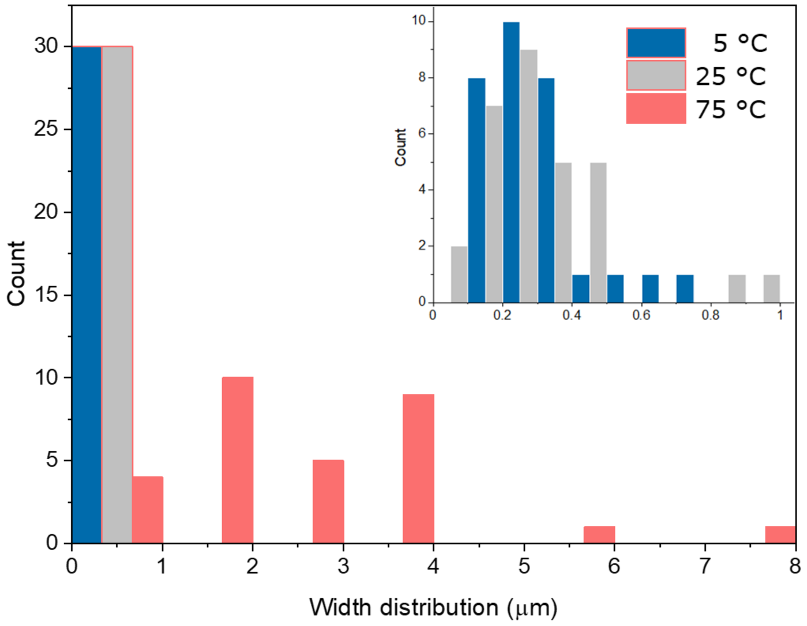

Figure S6. Width distribution of **DD(s)** prepared at different temperatures (inset: enlarged diagrams for 5 °C and 25 °C).

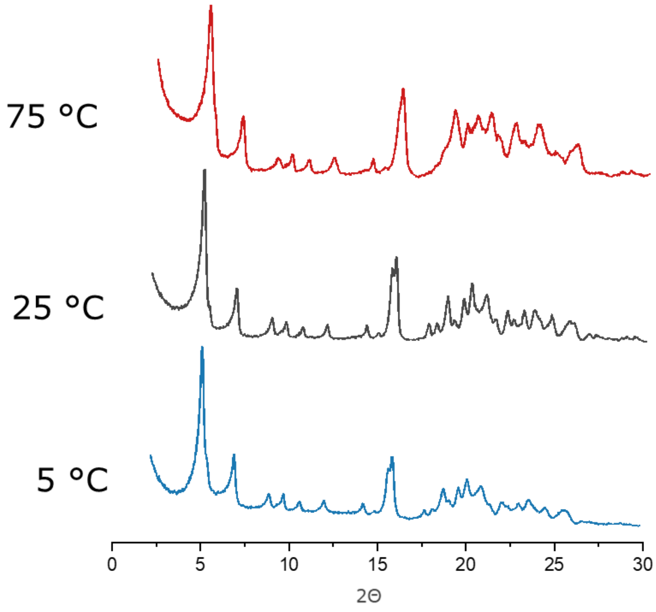

Figure S7. XRD profiles of **DD(s)** prepared at different temperature, confirming that the three compounds have the same crystallinity.

8. Chirality analysis

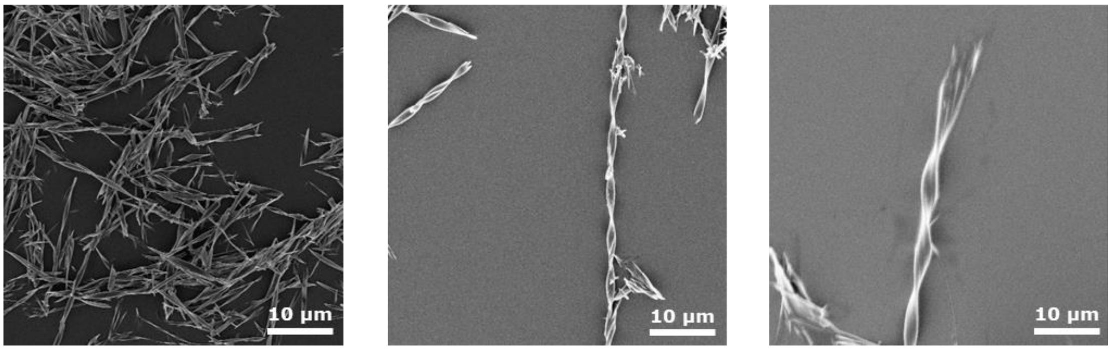

Figure S8. SEM images of **DD(s)** prepared with different ratio of HFIP to water (left: **DD(s)**, middle: **DD(s-5%)**, and right: **DD(s-10%)**).

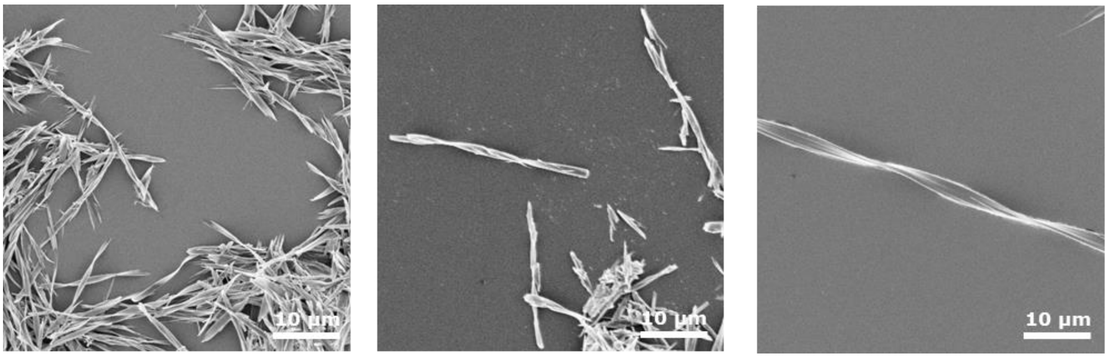

Figure S9. SEM images of **LL(s)** prepared with different ratio of HFIP to water (left: **LL(s)**, middle: **LL(s-5%)**, and right: **LL(s-10%)**).

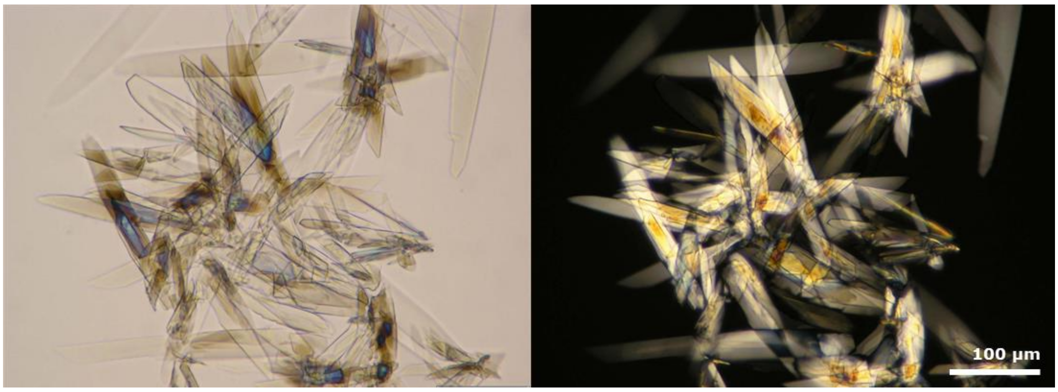

Figure S10. POM images of **DD-LL(s)** taken between parallel (left) and crossed (right) polarizers.

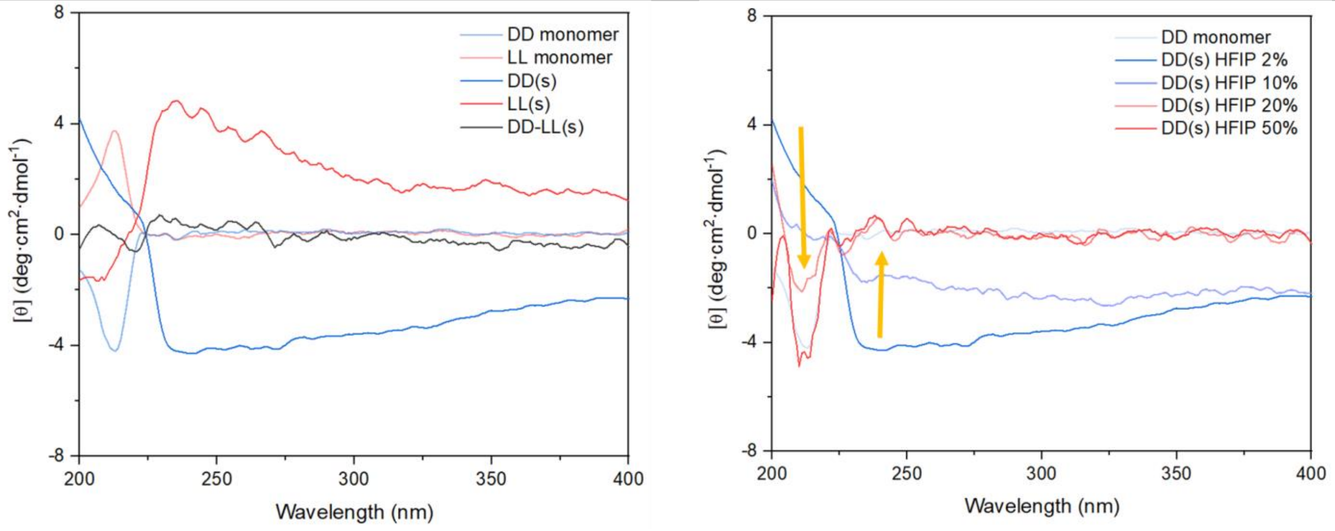

Figure S11. CD spectra of the disaccharides (**DD** and **LL** monomers) and their aggregates (**DD(s)** and **LL(s)**) (left). The CD spectra recorded for **DD** and **LL** monomers (300 µM in HFIP) show a peak at 212 nm, with opposite sign for the two enantiomers. Addition of water triggers the formation of helical fibers, as shown by the different CD spectra obtained for **DD(s)** and **LL(s)**, with a maximum at 234 nm. No CD signal is observed for the coassembly, **DD-LL(s)**, suggesting absence of supramolecular chirality. Disruption of the helical fiber and transition to the monomer state is monitored upon sequential addition of HFIP to **DD(s)** (right).

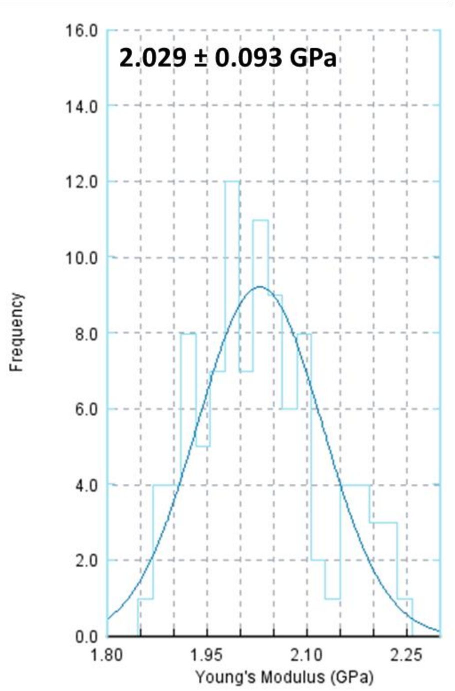

Figure S12. Young’s modulus measured for **DD-LL(s)** at a constant force of 800 nN.

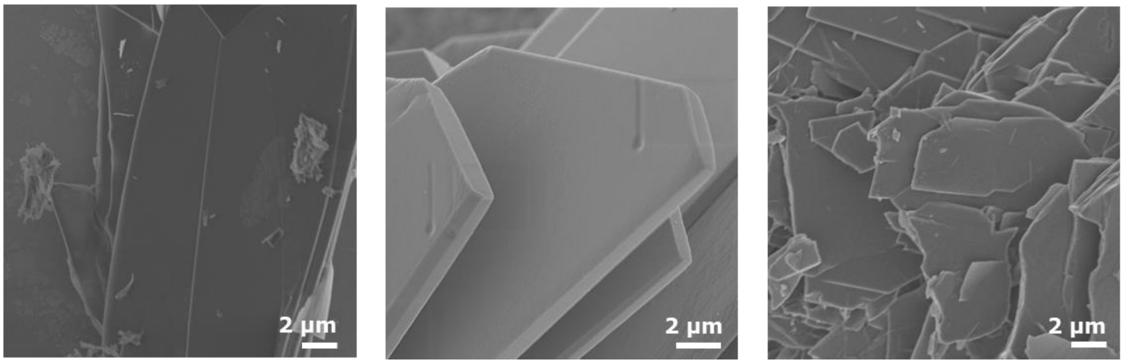

Figure S13. SEM images of **DD-LL(s)** prepared with different **DD:LL** ratios (left: 2:1, middle: 1:1, and right: 1:2).

9. 2D self-assembly

The samples were prepare as described in TableS2 *(Section 3)*.

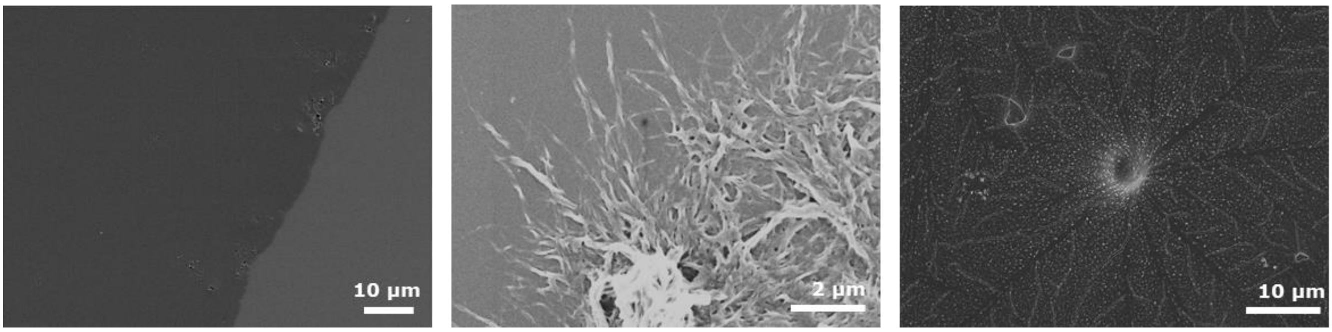

Figure S14. SEM images showing morphology transition on a thin film of Compound **DD**. Compound **DD** in HFIP generated a continuous film by simple evaporation (left). The film is highly hydrophobic. Upon direct contact with water, a fibrous structure is generated (middle). Similarly, the film incubated in a humidity chamber shows the transition to a crystalline structure (right, **DD(fr)**).

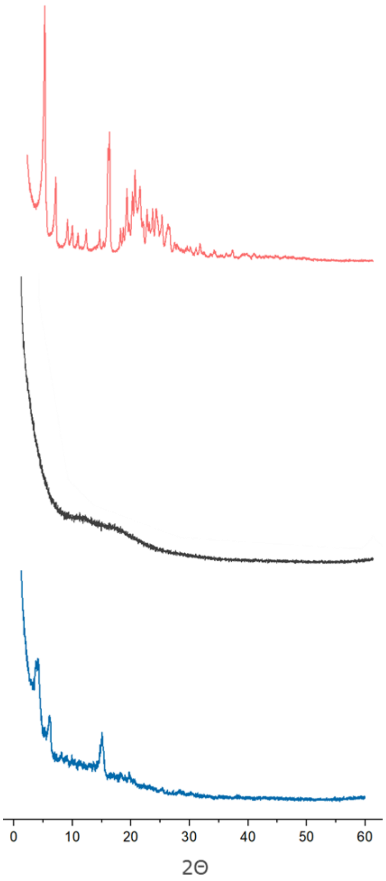

Figure S15. XRD profiles of **DD(s)** (red), **DD(fr)** (t = 0, black) and **DD(fr)** (t = 3 h, blue). The sample was directly prepared on the XRD sample holder, zero diffraction silicon. The **DD(fr)** (t = 0) is amorphous, showing a low intensity broad peak between 5° and 25°. Upon rehydration **DD(fr)** (t = 3 h) develops sharp peaks, identical to the peaks observed for the sample obtained upon solvent switch (**DD(s)**).

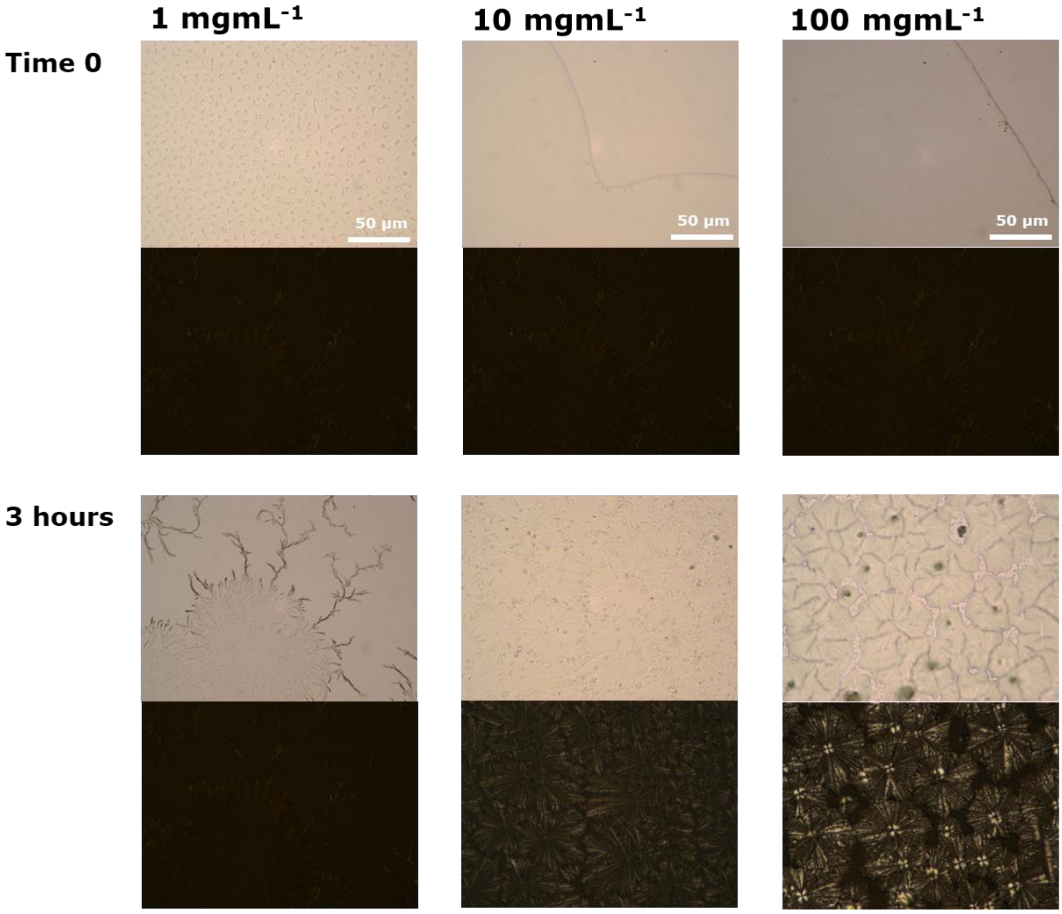

Figure S16. POM images of **DD(fr-1)**, **DD(fr-10)**, and **DD(fr)** at time 0 and after 3 hours in the hydration chamber. The samples are observed between parallel (top) and crossed (bottom) polarizers.

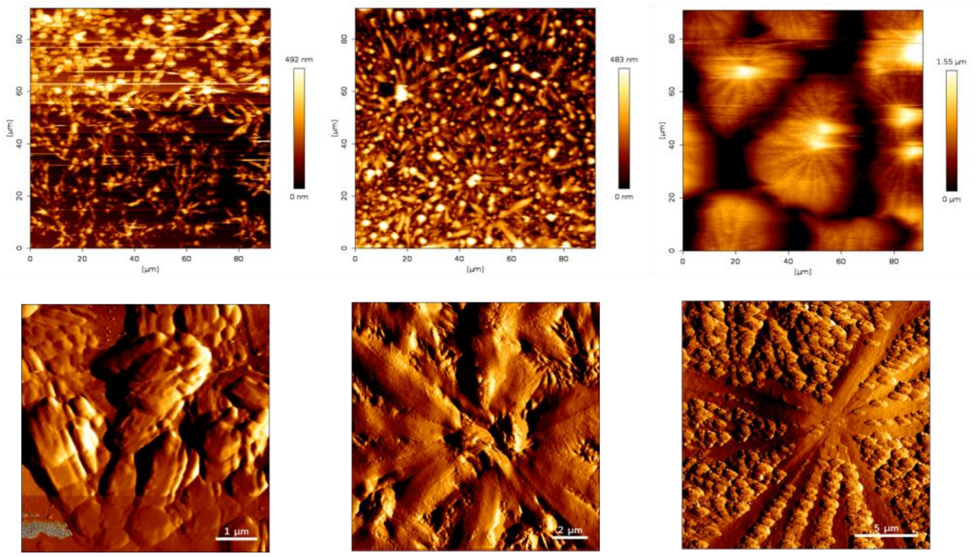

Figure S17. AFM height (top) and enlarged lock-in-amplitude (bottom) images of **DD(fr-1)** (left), **DD(fr-10)** (middle), and **DD(fr)** (right).

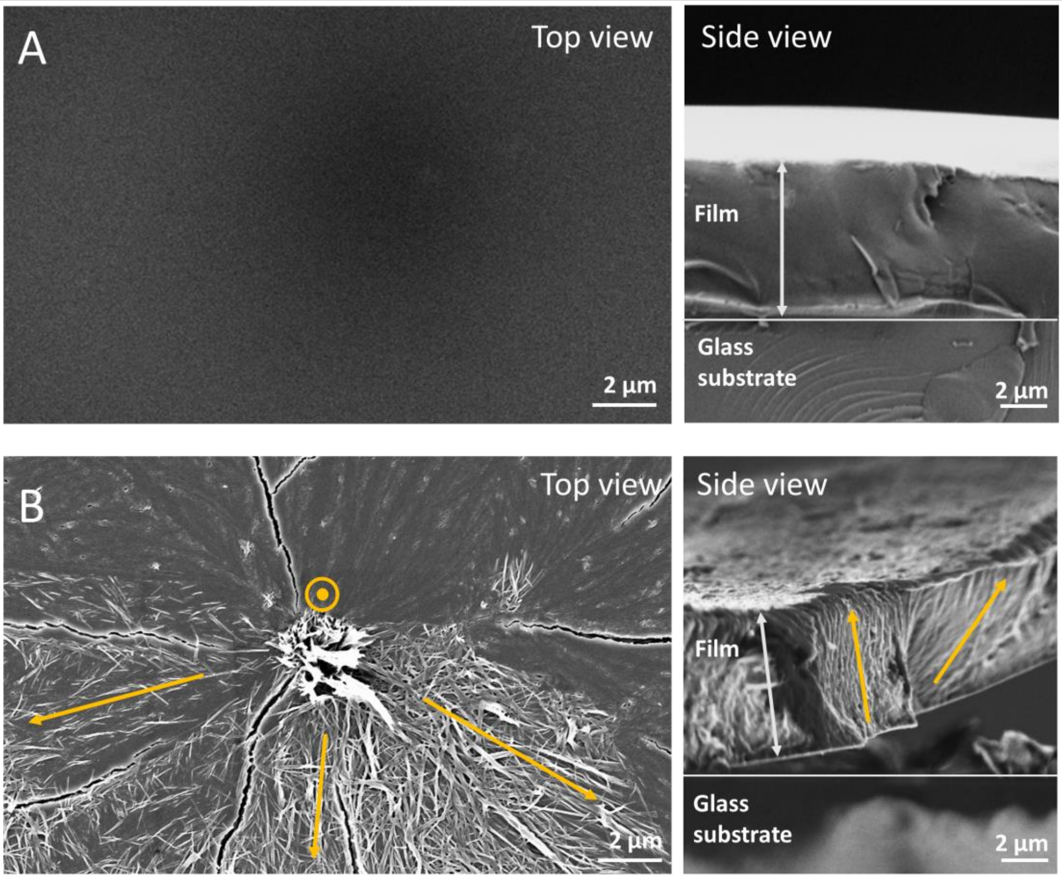

Figure S18. SEM cross-sectional images of **DD(fr)** before (A) and after (B) hydration. Upon hydration, the continuous film (A) turns into a fibrous structure (B).

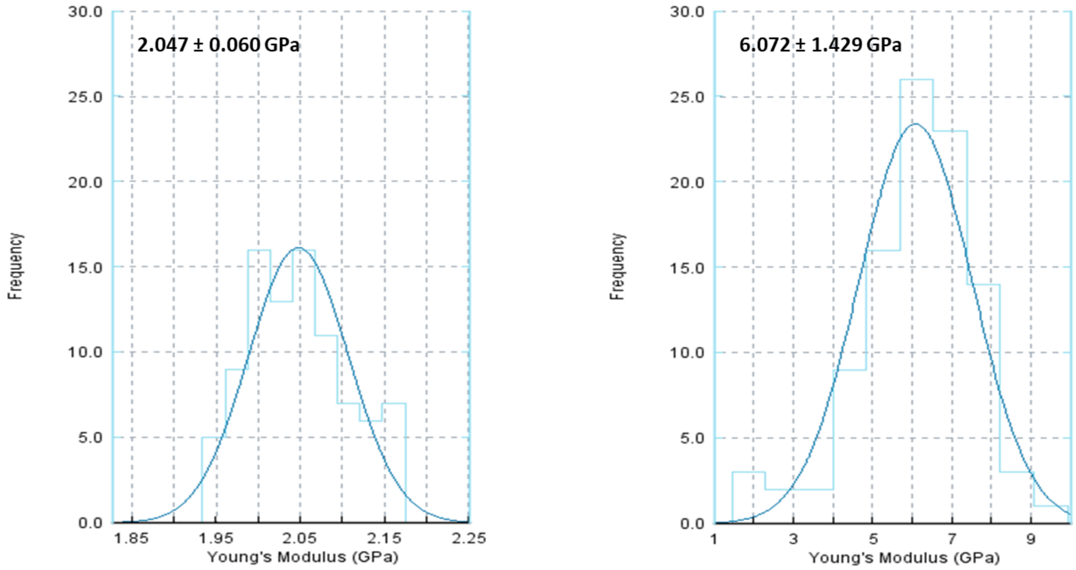

Figure S19. Young’s modulus measured for **DD(fr)** at t = 0 (no hydration, left) and at t = 3 h (right) at a constant force of 800 nN.

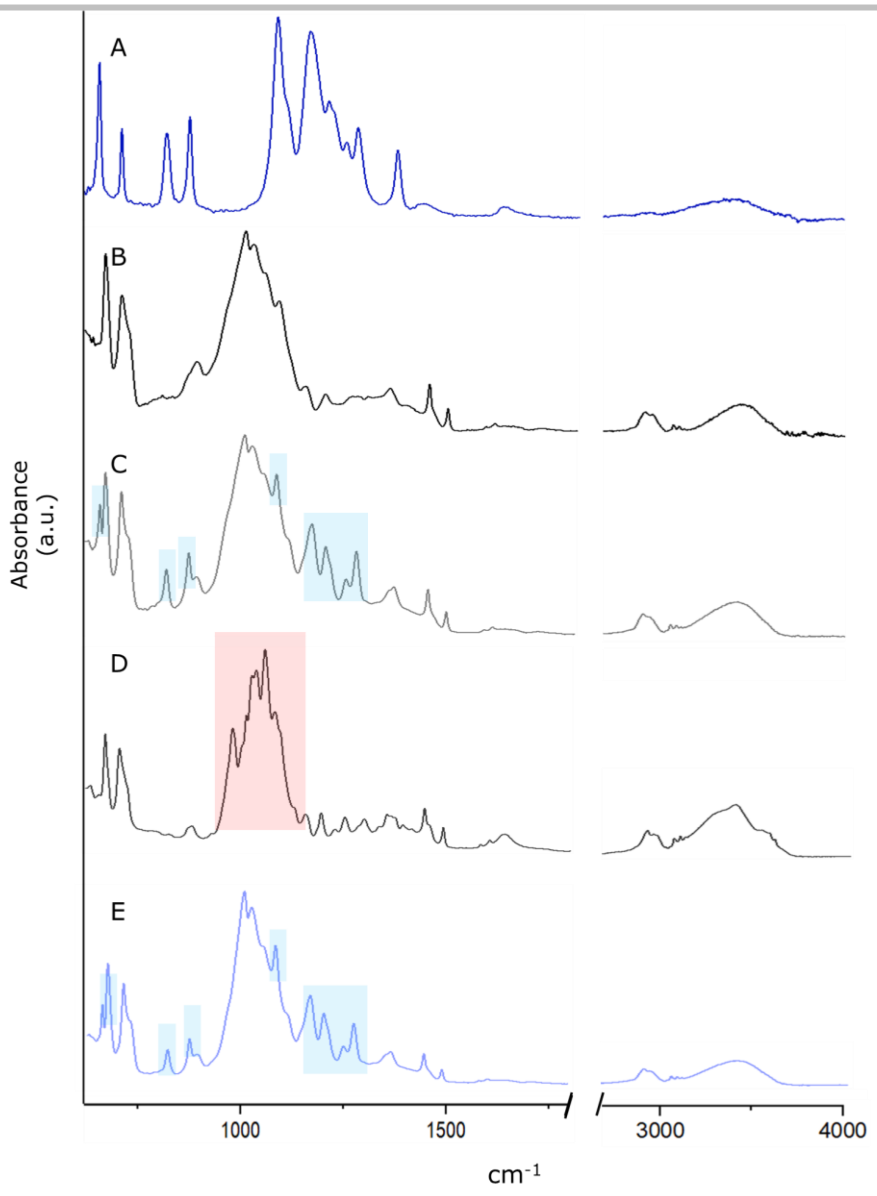

Figure S20. ATR-FTIR spectra of HFIP (A), **DD** powder as synthesized (B), **DD(fr)** at time 0 (C) and after 2 hours (D), and **DD(fr)** treated with HFIP vapor for 2 hours (E). Peaks highlighted with blue boxes in (C) indicate the presence of HFIP in the film. The peaks disappeared in (D) showing HFIP depletion upon incubation in water vapor.[4] The spectrum recorded between 998 and 1100 cm^{-1} in (D), highlighted with a red box, develop sharp peaks as an indication of molecular reorganization. Upon incubation of **DD(fr)** in HFIP vapor, the crystallized film returned to the amorphous state. The IR spectra (E) is identical to the one for **DD(fr)** at time 0, (C), showing the reversibility of the process.

10. References

[1] Y. Yu, S. Gim, D. Kim, Z. A. Arnon, E. Gazit, P. H. Seeberger, M. Delbianco, *J. Am. Chem. Soc.* **2019**, *141*, 4833-4838.

[2] J. Zielkiewicz, *J. Chem. Phys.* **2005**, *123*, 104501.

[3] D. Van Der Spoel, E. Lindahl, B. Hess, G. Groenhof, A. E. Mark, H. J. C. Berendsen, *J. Comput. Chem.* **2005**, *26*, 1701-1718.

[4] M. M. Pierre Chassagne, Nikolay Khanzhin, Markus Jondelius Hederos, **2017**, *WO2018077368A1*, May, 3, 2018.
